# Sitting and Active Meditation Practice: Utilization and Associations with Outcomes in Naturalistic and Clinical Trial Data

**DOI:** 10.1007/s12671-026-02787-w

**Published:** 2026-03-09

**Authors:** Simon B. Goldberg, Zishan Jiwani, Cortland J. Dahl, Raquel Tatar, John D. Dunne, Richard J. Davidson, Matthew J. Hirshberg

**Affiliations:** 1 Department of Counseling Psychology, University of Wisconsin–Madison, 335 Education Building, 1000 Bascom Mall, Madison, WI 53706 USA; 2 Center for Healthy Mind, University of Wisconsin – Madison, Madison, WI USA; 3 Humin, Madison, WI USA; 4 Department of Asian Languages and Cultures, University of Wisconsin – Madison, Madison, WI USA; 5 Department of Psychology, University of Wisconsin – Madison, Madison, WI USA

**Keywords:** Meditation, Informal practice, Mindfulness, Smartphone app, Mobile health

## Abstract

**Objectives:**

Digital technology opens the possibility of providing meditation instruction in the midst of daily activities. This study explores the use of “active” meditation practices which involve meditating while completing daily activities (e.g., folding laundry).

**Method:**

We used data from public users of the Healthy Minds Program (HMP) meditation app (*N* = 26,532, Sample 1) and from a recently completed trial testing the HMP app (*N* = 248, Sample 2). We examined associations between the proportion of practices completed as active practices (Active Proportion) with participant demographics, baseline psychological distress, patterns of app utilization, and changes in psychological distress.

**Results:**

Although sitting practice was used more commonly than active practices, active practices were frequently used (38% and 28% in Samples 1 and 2, respectively). Identifying as a woman or other gender was associated with a higher Proportion Active in Sample 1 (but not Sample 2). Associations with utilization differed across samples. Sample 1 showed a positive quadratic association where participants who primarily used active or sitting practices, rather than a combination, showed greater utilization. The opposite pattern was observed in Sample 2 (i.e., negative quadratic). Results were fairly consistent across sensitivity analyses. Use of active practice was not associated with poorer clinical effects.

**Conclusions:**

Active practices are commonly used when offered as a viable form of meditation and may be non-inferior to sitting practices. Further research manipulating practice posture (i.e., sitting versus active) is warranted.

**Preregistration:**

The randomized controlled trial from which Sample 2 was drawn was preregistered through clinicaltrials.gov (https://clinicaltrials.gov/ct2/show/NCT04426318). All analyses reported here were not preregistered.

**Supplementary Information:**

The online version contains supplementary material available at 10.1007/s12671-026-02787-w.

Meditation has become increasingly popular in the United States (US) in the past several decades (Nahin et al., 2024; Wielgosz et al., 2019). In 2022, meditation was the most widely used complementary and alternative medicine practice with 17.3% of US adults practicing meditation in the past year (Nahin et al., 2024). Furthermore, evidence suggests that meditation may be produce beneficial effects on a range of psychological challenges including, but not limited to, stress (Koncz et al., 2021), mood disorders (Blanck et al., 2018), post-traumatic stress disorder (Hilton et al., 2017), chronic pain (Lin et al., 2022), and sleep disorders (Rusch et al., 2019).

Meditation practice is theoretically a central ingredient in meditation-based interventions. While widely studied meditation-based interventions like Mindfulness-Based Stress Reduction (MBSR; Kabat-Zinn, 1990) include content that moves beyond meditation practice (e.g., psychoeducation regarding stress), participants are encouraged to invest heavily in regular meditation practice. The original recommendation within MBSR was 30–45 min per day of formal meditation practices, such as body scans or sitting meditation (Kabat-Zinn, 1982). There is meta-analytic evidence suggesting that at least within studies testing MBSR and Mindfulness-Based Cognitive Therapy (MBCT; Segal et al., 2013), participants do report completing a substantial amount of formal meditation practice at home (~ 30 min, 6 days per week) and that amount of home practice is modestly positively associated with benefit (*r* = 0.26; Parsons et al., 2017).

MBSR, MBCT, and other meditation-based interventions commonly emphasize what has been defined as informal meditation practice alongside formal practice. In the original MBSR manual, Kabat-Zinn (1990) defines formal practice to include the body scan, sitting meditation, and walking meditation, as well as basic hatha yoga poses (e.g., standing). Kabat-Zinn uses breathing meditation—a foundational practice in MBSR—to contrast formal and informal practice in the MBSR manual. Kabat-Zinn characterizes formal mindfulness of breathing practice as “the formal discipline of making a specific time in which you stop all activity, assume a special posture, and dwell for some time in moment-to-moment awareness of the inbreath and the outbreath” (p. 57). This is contrasted with informal mindfulness of breathing practice, which is characterized as “using the breath to be mindful of it from time to time during the day, or even all day long, wherever you are and whatever you are doing” (p. 57). Several key features highlighted in Kabat-Zinn’s characterization of formal practice include (a) time boundaries (i.e., a specific time), (b) a primary intention of practicing meditation during that period, and (c) a clear practice target (i.e., moment-to-moment awareness of the breath). In contrast, informal practice is (a) not time bound and (b) secondary to the activities of daily life. Crane et al. (2017) closely follow Kabat-Zinn’s distinction, describing formal meditation practice in mindfulness-based interventions as the body scan, mindful movement, and sitting meditation, and informal practice as “bringing awareness in particular ways to everyday activities” (p. 994).

Informal meditation practice is strongly emphasized in MBSR and many other meditation-based interventions as a means of bringing the same qualities developed through formal practice more fully into daily life, with Kabat-Zinn (1990) describing informal practice as “at least as valuable as the formal practice” (p. 57). Informal practice has deep Buddhist roots as well, with the three major Buddhist traditions—Zen, Tibetan, and Theravadan—all emphasizing the value in applying meditative techniques in the context of daily life activities. For example, the *Satipaṭṭhāna Sutta* (*sati* denoting mindfulness in Pali) encourages the development of mindfulness of the body throughout a wide variety of daily life activities (e.g., “when going forward and returning…when eating, drinking,…falling asleep, waking up, talking, and keeping silent,” Anālayo, 2006, p. 5).

Although informal practice has received far less scientific attention than formal practice, several randomized trials have examined the effects of informal practice, with results supporting its potential therapeutic benefit. For example, Hanley et al. (2015) randomized 51 university students to receive instructions in mindful dishwashing or to a control group that received a general description of washing dishes. Participants in the mindful dishwashing condition reported higher state mindfulness along with increases in positive affect and decreases in negative affect relative to the control group. In a study with 226 university students, Petrovic et al. (2025) found that brief informal mindfulness practice—but not formal mindfulness practice—was preferred over a control task (copying letters, numbers, and symbols into a grid). Moreover, while both informal and formal mindfulness practice increased state mindfulness, only informal mindfulness decreased stress relative to the control condition. Shankland et al. (2021) investigated a group-based mindfulness intervention that included only brief and informal practices aimed at increasing awareness in daily activities. In a waitlist controlled randomized trial (*n* = 139), they reported benefits on measures of psychological distress and well-being that persisted several months after the 8-week intervention. Alongside these randomized trials, several studies have demonstrated linkages between informal practice in daily life and improvement on markers of distress and well-being (e.g., Manigault et al., 2021; Xie et al., 2024).

The delivery of meditation practice through digital technology has the potential to further enhance the integration of meditative techniques within daily life (Creswell & Goldberg, 2025), thereby sharing an aim with informal practice. Meditation apps are by far the most widely used digital mental health interventions (Wasil et al., 2020) and the smartphones that deliver these interventions are often within arm’s reach of most individuals across the globe (Taylor & Silver, 2019). The current study examines a form of meditation practice instruction—what we call active practices—that are designed to be completed amid simple daily life activities such as when commuting or folding laundry. Instruction in these daily life contexts is made possible through a meditation app, the Healthy Minds Program (HMP). Within HMP, active practices are contrasted with sitting practices which, like traditional formal practices in MBSR, are designed to be done during time set aside for an exclusive focus on practice (Kabat-Zinn, 1990).

Are active practices formal or informal meditation practice? Active practices share similarities with both informal and formal practice. In our view, the boundary between informal and formal practice is an area worthy of further conceptual development, with the distinction not necessarily being as clear as it may seem. While a thorough treatment of this issue is outside the current paper, some discussion of active practices in relation to both informal and formal practice is important before investigating it empirically.

Drawing from sociology and anthropology, ritual theory provides a useful framework for understanding definitional boundaries between formal and informal practices, as well as the complexity of drawing such boundaries. Characterizing precisely what does and does not constitute ritual has proved challenging for sociologists, anthropologists, and other scholars for decades (e.g., Goody, 1961). In the book *Ritual Theory, Ritual Practice*, Bell (1991) emphasizes “‘ritualization’ as a strategic way of acting,” (p. 7) where “acting ritually emerges as a particular cultural strategy of differentiation” (p. 8). Bell acknowledges, however, that “attempts to distinguish clearly between rite and non-rite” are “perennial obstacles to neat definitions and classification” (p. 8). In a recent review emphasizing the psychology of rituals, Hobson et al. (2018) define them to include three key elements: “(a) predefined sequences characterized by rigidity, formality, and repetition that are (b) embedded in a larger system of symbolism and meaning, but (c) contain elements that lack direct instrumental purpose” (p. 261). They highlight that although rituals include all three, their relative weight may differ. Hobson et al.’s definition of ritual is intentionally broad, designed to capture both religious rituals as well as psychological symptoms that involve ritualistic behavior (e.g., in obsessive–compulsive disorder).

Applied to meditation practice, we might fairly neatly categorize formal meditation practice as formal based on it meeting the criteria outlined for a ritual: a rigid, predefined sequence (e.g., an MBSR participant lays down on their yoga mat to engage in a body scan) that is embedded in a larger meaning system (e.g., this is a practice from the MBSR course where they are developing their mindfulness skills) that contains elements that lack direct instrumental purpose (e.g., there is not another task being accomplished while they lay on the yoga mat aside from mental training). An informal meditation practice, for example, attempting to bring mindfulness to the activity of washing the dishes, does not necessarily meet these criteria. One would expect less formality and rigidity during this activity, and, although it may be embedded within a larger meaning system (e.g., washing the dishes mindfully is a way to develop mindfulness skills in daily life), it does not contain elements without direct instrumental purpose, perhaps aside from the mental practice of attempting to maintain mindful awareness during the activity.

There are clear Buddhist origins for the distinction between formal and informal practice as well. In Tibetan Buddhism, for example, the term *thun* (pronounced “toon”) refers to a meditation practice “session” that contrasts with *thun mtshams* (pronounced “toon-tsam”), which refers to the “between session” period. Tibetan Buddhism makes a psychological distinction as well, using the term *gnyam bzhag* (“nyam-shak”) to refer to the meditative “state” and *rjes thob* (“jay-tōp”) as the “post-state,” that, although “perfumed” by the state (Bkra-shis-rnam-rgyal, 2019), is nonetheless psychologically distinct. All three major Buddhist traditions (Zen, Tibetan, Theravadan) have formal practice rituals that meet Hobson et al.’s (2018) definition, notably with very clear start and end times (e.g., meditation sessions signaled through bells or gongs; Kapleau, 2013). All three major Buddhist traditions also offer what we might define as formal practices that meet the definition of ritual but do not involve sitting still. Examples include walking meditation, chanting, and prostrations, all of which may be highly rigid, embedded within broader meaning systems, and containing elements that lack instrumental purpose (Hobson et al., 2018).

However, as has plagued scholars seeking to differentiate rite from non-rite (Goody, 1961), there are Buddhist traditions that very intentionally blur the line between formal and informal practice. In the nondual Tibetan traditions, for example, a meditative goal for the most advanced practitioners is being able to maintain the meditative state even while “between sessions.” Advanced meditation practitioners may shift from engaging in formal meditation in more structured, cloistered environments to engaging in meditative practice immersed in the context of daily (e.g., completing wandering retreats; Mingyur Rinpoche, 2019). While users of a a meditation app are unlikely to be at this advanced level, it is nevertheless important to note that this theoretical blurring of the lines between a formal session and “between sessions” is clearly articulated in certain Tibetan traditions and has implication for practice even by novices.

Active practices implemented in the HMP app are inspired by the Tibetan Buddhist traditions that have intentionally blurred the line between formal and informal practice, albeit at advanced levels of practice (e.g., Bkra-shis-rnam-rgyal, 2019). Like sitting practices, active practices in HMP involve sessions lasting a predetermined length (giving them a more rigid and predefined sequence) with a clearly defined script that is situated within a broader intervention framework (i.e., system of meaning), meeting the first and second criteria by Hobson et al. (2018). However, unlike typical formal practice which makes no direct instrumental contribution to an ordinary activities (such as washing the dishes), these practices are explicitly intended to be done while engaging in such activities. At the same time, the practice script prominently includes mental instructions that do not themselves have a direct instrumental purpose toward any daily activity. In this way, active practices are not easily characterized by the binary distinction between formal and informal practice, as defined in MBSR (Kabat-Zinn, 1990). If, however, we consider “formal/informal” to be a spectrum rather than a rigid binary distinction, then we might place active practices closer to the “formal” end of that scale. But because they involve deliberate engagement with daily activities, active practices may have effects or mechanisms that resemble informal practices, at least as they have been conceived and studied in the literature thus far.

To our knowledge, no research has yet examined active practices as implemented in HMP. Yet, there are several potential benefits to active meditation as a supplement to sitting meditation and even as a primary practice posture that make them worthy of study. First, findings from cognitive science suggest that learning a new skill may be aided by applying that skill in a variety of settings and contexts (i.e., experiential learning; Gorghiu & Santi, 2016). Given its flexibility, active meditation can be easily done in a range of contexts. This is particularly important because skill development by repetition within a narrow domain may fail to transfer outside of the narrow domain of practice (Singley & Anderson, 1989), limiting real-world utility. Thus, active practices may help support transfer learning for self-regulatory skills developed during meditation (Schuman-Olivier et al., 2020). Second, it is possible that active meditation may be more accessible, at least for some populations. Formal sitting meditation practice is a known barrier for some populations (e.g., Petrovic et al., 2025). Research suggests that meditation and other well-being practices are more frequently accessed by higher socioeconomic groups (Jiwani et al., 2023; Macinko & Upchurch, 2019; Olano et al., 2015). One driver of this disparity may be that individuals of lower socioeconomic status have more limited time to engage in formal sitting practice (Blum, 2014). Additionally, other groups such as parents of young children or other caregivers may also struggle to find time for sitting practice (Leitch et al., 2023; Murfield et al., 2021). Active practice may facilitate engaging in formal practice while also being present for their responsibilities.

While active practice may have several potential advantages, less is known about whether it is as efficacious as sitting practice. The existing literature suggests that formal walking and eating meditation practice may produce benefits, at least in some instances (e.g., on mental health, blood glucose levels, and proprioception; Gainey et al., 2016; Lapanantasin et al., 2022; Prakhinkit et al., 2014; Shi et al., 2019) and informal practice also has shown promise (as discussed above, e.g., Hanley et al., 2015). However, active meditation as we are describing it here—in some ways a hybrid of formal and informal practice—has not been studied directly. Many basic questions about active practice remain unanswered. It is unclear (a) the degree to which individuals will engage with active practice when it is offered as a viable form of meditation practice, (b) whether demographic characteristics or psychological distress are associated with use of active practices, (c) whether engagement with active practices is associated with different patterns of persistence with meditation practice (i.e., do active practices make practice more accessible), (d) whether engagement with active practices is associated with changes in psychological outcomes in response to meditation training.

To answer these questions, we used data from a meditation app—the HMP—that allows users to select from both sitting and active practices when using guided meditations. We gathered data from public users of HMP as well as data from a recently randomized controlled trial testing HMP in public school employees (Hirshberg et al., 2022). We sought to examine naturalistic patterns of use to establish the following: (1) how much sitting or active meditation is used when both are offered as viable forms of meditation practice, (2) demographic and psychological distress correlates of sitting versus active meditation, and (3) whether patterns of utilization or changes in psychological outcomes vary based on the proportion of sitting versus active meditation.

## Method

### Participants

The present study used two distinct samples, both of which accessed HMP. The first sample (Sample 1) consisted of public users of the freely available HMP app between September 2019 and July 2022. The second sample (Sample 2) included the intervention arm of a randomized controlled trial assessing the efficacy of the HMP app in school system employees during the COVID-19 pandemic with recruitment occurring between June and August 2020 (Hirshberg et al., 2022).

For Sample 1, initially, 65,755 unique HMP users were identified who had downloaded the app from a US Apple or Google app store during the study period and reported at least one demographic variable. Participants (*N* = 26,532, 40.3%) were included if they engaged in at least two practice sessions. We restricted the sample to those who engaged in at least two sessions as this would allow individuals to potentially explore both sitting and active meditation. We examined the first 30 days of use in our primary analyses, given this is a critical period for engaging with mobile apps (Baumel et al., 2019). Participants who first engaged with the app less than 30 days before the data collection was completed (i.e., after June 2022) were excluded as they did not have 30 days of usage to analyze. Participants agreed to share their deidentified data for research purposes as part of the HMP app terms and conditions.

Sample 2 was drawn from a randomized controlled trial conducted during the COVID-19 pandemic where school system employees in the state of Wisconsin were either assigned to HMP or a waitlist control. Complete methods and results of the study are reported elsewhere (Hirshberg et al., 2022). Participants (*n* = 248, 72.1% of those randomized to HMP) were included if they engaged in at least two practices. App usage and self-report data were collected in the Summer and Fall of 2020 and included assessments prior to the start of the intervention (T1), at post-intervention (T2), and 3 months after the end of the intervention (T3). Participants provided a written informed consent to participate in the study.

### Procedure

The HMP app has demonstrated efficacy in alleviating symptoms of psychological distress in randomized controlled trials (Goldberg et al., 2020a, 2020b; Hirshberg et al., 2022). The app is built around four modules based on the components of well-being proposed by Dahl et al. (2020): awareness, connection, insight, and purpose. Each module includes psychoeducation and meditation practices focused on each component of well-being. Awareness practices are designed to cultivate mindfulness (i.e., non-judgmental, present-moment attention) and meta-awareness. Connection practices are designed to cultivate healthy relationships with oneself and others (e.g., through kindness or compassion-style practices). Insight practices are designed to cultivate self-knowledge and insight into patterns of thoughts, feelings, and behaviors. Purpose practices are designed to cultivate clear connections between one’s daily activities and broader values in one’s life. Participants are introduced to both sitting and active practices during the initial app content. All guided practices included in the app allow users to select between sitting or active practices that are equivalent in duration. A sample script for sitting and active practice is provided in Table 1.

**Table 1:**
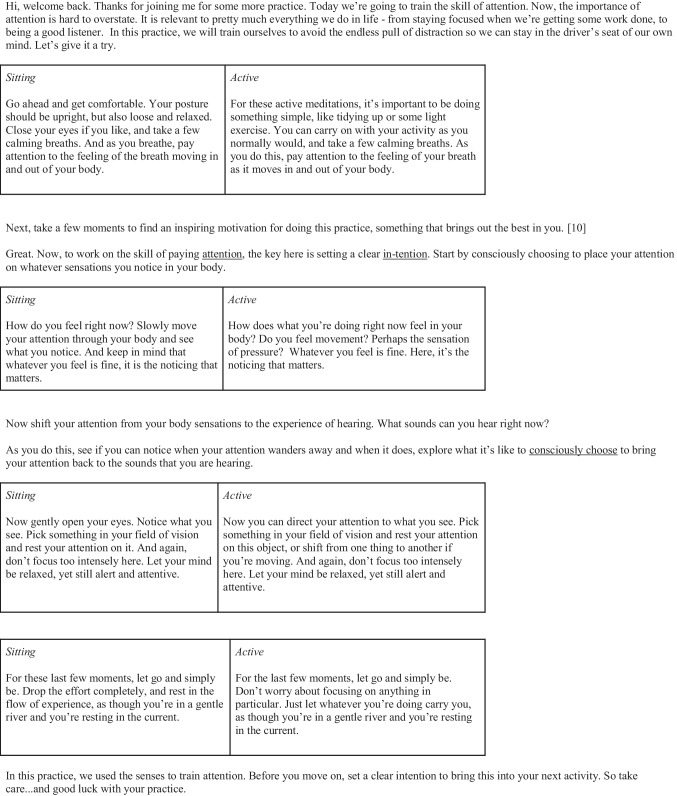
Sample guided meditation practice with instructions for sitting and active style

### Measures

#### Utilization Metrics

All utilization metrics were gathered objectively through HMP. HMP utilization was operationalized as the total number of meditation practice sessions a participant completed (*Sum Practice*) and the total number of days a participant utilized the app (*Sum Days*) within first the 30 days. *Sitting Practice* was the total number of meditation sessions where the participant chose the sitting practice type. *Active Practice* was the total number of meditation sessions where the participant chose active practice type. *Active Proportion* was a measure of the proportion of meditation sessions which were active (i.e., the number of active practices divided by the total number of practices). Practice variables (i.e., Sum Practice, Sitting Practice, etc.) did not include didactic components of the app.

#### Demographic Measures

Demographic variables included in Sample 1 were age, race/ethnicity, gender, education, and relationship status. All variables, including continuous variables, were collected in the app categorically. For age, there was a shift in the categories over time (e.g., the 19–24 category was later changed to 18–24). Age 34 was a consistent cut point across categorical changes and prior epidemiologic work investigating access to mental health treatment has used 34 as a cut point for a younger age (Mojtabai et al., 2011). Thus, we dichotomized age into 34 and younger or 35 and older. We adapted the race/ethnicity variable from the structure proposed by the US Census Bureau (2022). Six racial/ethnic groups were included: (1) Non-Latine (NL) White; (2) Black or African American; (3) Latine; (4) Asian; (5) Native American, Hawaiian, or Other Pacific Islander; and (6) Other (i.e., “none of the above fully describe me”). The US Census also included a multiracial category. However, this category was not available as an option for app users and thus was not included in the present sample. Gender was categorized into:\ Woman, Man, and Other. The Other category encompassed individuals identifying as gender non-binary, gender fluid, or other genders. The education variable was dichotomized between those with a college degree (1) and those without a college degree (0) consistent with prior research indicating that college completion is an important marker of differentiation within the HMP app (Jiwani et al., 2023). Marital status was dichotomized between those currently married or in a domestic partnership (1) and those who did not describe themselves as either married or in a domestic partnership (0) for ease of interpretation.

Demographic variables included in Sample 2 were age, race/ethnicity, gender, education, income, and relationship status. Age was collected and included as a continuous variable. As the sample was predominantly NL White (*n* = 223, 89.9%) and relatively small, we dichotomized race as NL White and all other groups. For gender, participants predominantly identified as women (*n* = 221, 89.1%). The sample was dichotomized as women and men as no other genders were reported. Education and marital status were dichotomized as described above due to the small cell sizes. Income was collected categorically and included the following categories: less than $50,000, $50,000–$100,000, $100,000–$150,000, and greater than $150,000.

#### Psychological Distress

Psychological distress was assessed only in Sample 2. We operationalized psychological distress as the composite of depression, anxiety, and stress measures, which was the trial’s preregistered primary outcome (Hirshberg et al., 2022). Computer-adaptive versions (Version 1.0) of the Patient Reported Outcomes Measurement Information Systems (PROMIS) Depression and Anxiety measures (Pilkonis et al., 2011) were used to assess depression and anxiety symptoms. Both measures have demonstrated strong convergent validity with legacy measures (Choi et al., 2014; Schalet et al., 2014). Items assessed symptoms of depression (“I felt helpless”) and anxiety (“My worries overwhelmed me”) over the past 7 days on a 5-point scale from 1 (*never*) to 5 (*always*). A *T*-score was derived from the computer adaptive versions (i.e., population: mean [*M*] = 50, standard deviation [*SD*] = 10). As participants do not receive the same set of items, internal consistency cannot be assessed for the computer adaptive versions of the PROMIS. However, prior fixed form versions have demonstrated good internal consistency (Pilkonis et al., 2011). The 10-item NIH Perceived Stress Scale (PSS, S. Cohen et al., 1983; Cyranowski et al., 2013) was utilized to assess psychological stress. PSS examines stress-related experiences in the past month (e.g., “How often have you felt that you were unable to control the important things in your life?”) with items rated on a 5-point scale from 1 (*never*) to 5 (*very often*). A total score was computed by summing all PSS items (McDonald’s *ω* =.89). A composite psychological distress measure was computed by *z*-scoring each of the three outcomes and then calculating an average of the three *z*-scores (*w* =.87). We also included a sensitivity analysis that examined effects on depression, anxiety, and stress separately.

### Data Analyses

Analyses for both samples were performed using R (R Core Team, 2025). Cohen’s (1992) guidelines were utilized to assess the magnitude of effect sizes. Standardized betas were calculated using the *lm.beta* function (Behrendt, 2022) in R for all linear regression models. We examined how much sitting or active meditation is used when both are offered using paired sample *t*-tests. As a descriptive statistic, we compared the mean number of meditation practice sessions and days of app utilization across the two samples.

To evaluate demographic correlates of sitting versus active meditation for Sample 1, we examined whether demographic characteristics were associated with active proportion using multiple linear regression models. The following demographic characteristics were used as the reference group: NL White, non-college educated, man, unmarried/divorced, and age greater than 34. Given the inclusion of five demographic categories, we utilized a Bonferroni correction to reduce the likelihood of type I error. Thus, we only interpreted the results of demographic comparisons with two-tailed *p* < 0.010 (i.e., 0.05/5). No further *p*-value adjustments were made. We conducted several sensitivity analyses designed to evaluate whether the patterns of findings differed based on methodological choices. Specifically, we assessed if the associations were consistent when we limited the sample to individuals who engaged in at least 3, 4, and 10 practices. This was done as the proportion of active practices, which was our primary outcome of interest, could be heavily influenced by a single active practice when participants with only two practices were included. We also assessed if the results were consistent if we limited app engagement to the first 7 days of use. This was done to assess any impact of including a longer period of observation (i.e., 30 days). In addition, we examined whether associations between demographics and proportion of active practices differed when covarying the total days of engagement, given participants had a range of engagement with HMP.

We used regression to address whether utilization (Sum Practice, Sum Days) varied based on the proportion of active meditation. Sum Practice deviated substantially from normality (skew = 4.11, kurtosis = 31.38; (Curran et al., 1996)) and thus was winsorized to two standard deviations (Tukey, 1962). We initially conducted exploratory data analysis (Tukey, 1977) in order to understand potential relationships between use of active practices and utilization of the HMP app using data visualization. As shown in Supplemental Figs. 1 and 2, visualizations included a linear and a local regression (i.e., loess; Jacoby, 2000) curve. The local regression curve suggested that the relationship between utilization and Active Proportion followed a non-linear pattern. We then tested regression models that included linear, quadratic, and cubic terms. Although a significant cubic term was found in some models, the quadratic models fit the data better across sensitivity analyses (Supplemental Table 1). Thus, we utilized the quadratic model as our final model which included linear and quadratic transformations of Active Proportion and demographic variables as independent variables with Sum Practice or Sum Days as dependent variables. We ran three sets of sensitivity analyses to assess if the results were robust. We examined the impact of limiting the sample to individuals who engaged in at least three, four, and ten practices; calculated app engagement metrics over the first 7 days of use; and examined results using the raw rather than winsorized version of Sum Practice.

In Sample 2, as for Sample 1, we used multiple linear regression to examine demographic and psychological distress correlates of sitting versus active meditation. The following demographic characteristics were used as the reference group: NL White, non-college educated, man, unmarried/divorced, and income of $50,000 or less. Psychological distress at T1 was also included in the model. Given the inclusion of seven variables, we utilized a Bonferroni correction to reduce the likelihood of type I error. As such, we only interpreted results of this model with two-tailed *p*s < 0.007 (i.e., 0.050/7). No further *p*-value adjustments were made. As with Sample 1, in sensitivity analyses, we assessed if the associations were consistent when limiting the sample to individuals who engaged in at least three, four, and ten practices and whether results were consistent if we limited app engagement to the first 7 days of use. As in Sample 1, we examined whether these associations differed when covarying the total days of engagement.

To examine whether utilization or psychological outcomes varied based on the proportion of active meditation, we used Sum Practice and Sum Days as the utilization outcomes and psychological distress at T2 and T3 as the psychological outcomes (with T1 psychological distress as a covariate). Utilization and psychological distress variables did not deviate beyond recommended cutoffs for normality in this sample (i.e., skewness < 2, kurtosis < 7; (Curran et al., 1996)). Thus, we did not perform any transformations. As in Sample 1, we conducted exploratory data analysis (Tukey, 1977) through data visualization. As shown in Supplemental Figs. 3, 4, 5 and 6, visualizations included a linear and a local regression (i.e., loess) curve. The local regression curve suggested that the relationship between utilization as well as changes in psychological distress and Active Proportion was non-linear. We then tested regression models that included linear, quadratic, and cubic terms. The quadratic but not the cubic term was significant across the models (Supplemental Table 2). Thus, we utilized the quadratic model as our final model which included linear and quadratic transformations of Active Proportion along with demographic variables and T1 Psychological Distress as independent variables. Sum Practice, Sum Days, T2 Psychological Distress, and T3 Psychological Distress served as the dependent variables. We ran the same sensitivity analyses described above for Sample 1.

With regard to model assumptions, for Sample 1, prior methodological work indicates that linear regression estimates and inference are robust to moderate violations of distributional and variance assumptions in very large samples (Lumley et al., 2002). Given that Sample 2 had a smaller sample size, we evaluated assumptions for the primary models using quantitative diagnostics following, which indicated negligible nonlinearity, residual skewness and kurtosis well within commonly cited acceptable ranges (West et al., 1995), no evidence of substantial heteroscedasticity (Hayes & Cai, 2007), and variance inflation factors below recommended thresholds, indicating no problematic multicollinearity (Marcoulides & Raykov, 2019; see Supplemental Table 3). In addition, the relative convergence of findings across a large number of sensitivity analyses provides further context for the robustness of the reported results, suggesting that any remaining assumption departures were unlikely to materially affect inference.

### Missing Data

Utilization data were never missing in either sample as utilization was collected passively through the HMP app. As participants had a choice whether to report each demographic variable, an unknown category was included for all demographic variables. Pairwise deletion (i.e., complete case analysis) was used in models that included psychological distress.

## Results

Descriptive statistics for Sample 1 are reported in Table 2 and for Sample 2 are reported in Table 3. Individuals in Sample 1 (*N* = 26,532) predominantly identified as women (*n* = 17,492; 65.9%), college educated (*n* = 16,984; 64.0%), and NL White (*n* = 18,097, 68.2%). Individuals in Sample 2 (*N* = 248) also predominantly identified as women (*n* = 221; 89.1%), college educated (*n* = 221, 89.1%), and NL White (*n* = 223, 89.3%). Mean number of meditation practice sessions (i.e., Sum Practice; *M* = 9.27, *SD* = 10.68) and days of app utilization (i.e., Sum Days; *M* = 8.57, *SD* = 6.92) for Sample 1 were lower relative to mean number of meditation practice sessions (*M* = 19.35, *SD* = 8.64; *β* =  − 0.09, *p* < 0.001) and days of app utilization (*M* = 14.96, *SD* = 7.37; *β* =  − 0.09, *p* < 0.001) in Sample 2. The Proportion Active in Sample 1 was 38.0% and in Sample 2 was 27.7%.

**Table 2:** Sample demographics and utilization metrics for Sample 1 (*N* = 26,532)

Variable	Mean	*SD*	*n*	%	Min	Max	Skew	Kurtosis
Sum Practice	9.27	10.68			2	224	4.15	35.33
Sum Practice (winsorized)	7.92	6.04			2	20.1	0.9	2.48
Sum Days	8.57	6.92			1	31	1.31	4.05
Sum Sitting	5.88	7.78			0	124	3.94	31.19
Sum Active	3.39	6.36			0	147	7.87	106.7
Active Proportion^a^	0.38	0.27			0	1	0.51	2.39
Used HMP on day 30^b^			4724	7.2%				
Age								
Above 34			17,933	67.6				
34 or less			8132	30.9				
Unknown			467	1.8				
Gender								
Man			8244	31.1				
Woman			17,492	65.9				
Other			397	1.5				
Unknown			301	1.5				
Education								
Non-college grad			6046	22.8				
College grad or higher			16,984	64.0				
Unknown			3502	13.2				
Marital status								
Unmarried			12,244	46.1				
Married			13,413	50.7				
Unknown			875	3.3				
Race and ethnicity								
NL White			18,097	68.2				
Black			994	3.7				
Latine			1703	6.4				
Asian			1409	5.3				
Native			190	0.7				
Other			681	2.6				
Unknown			3398	13.0				

*M*(SD) = mean (standard deviation); *Min* minimum, *Max* maximum, *NL White* Non-Latine White, *winsorized* winsorized to two standard deviations

^a^The Active Proportion when examining > 2 practices was 0.37, when examining > 3 practices was 0.36 and when examining > 9 practices was 0.35. When examining just the first 7 days of engagement for individuals who engaged in > 1 practice, Active Proportion was 0.40

^b^This percentage is based on the sample of HMP users who provided any demographic data (*N* = 65,755, i.e., prior to restricting to those with ≥ 2 practices)

**Table 3:** Sample demographics and utilization metrics for Sample 2 (*N* = 248)

Variable	Mean	*SD*	*n*	%	Min	Max	Skew	Kurtosis
Sum Practice	19.35	8.64			2	33	− 0.52	2.10
Sum Days	14.96	7.37			1	29	− 0.14	1.95
Sum Sitting	13.68	7.89			0	30	0.20	2.01
Sum Active	5.67	6.17			0	25	1.28	3.92
Active Proportion^a^	0.28	0.26			0	1	0.75	2.49
Used HMP on day 30			69	27.8				
T1 Psychological Distress^b^	0.02	0.86			− 2.58	2.03	− 0.24	2.79
T2 Psychological Distress^c^	− 0.62	0.89			− 3.31	1.82	− 0.15	3.07
T3 Psychological Distress^d^	− 0.50	0.83			− 2.69	1.70	0.02	2.74
Age	42.6	10.61			23	73	0.31	2.61
Gender								
Man			26	10.5				
Woman			221	89.1				
Unknown			1	0.4				
Education								
Non-college grad			26	10.5				
College grad or higher			221	89.1				
Unknown			1	0.4				
Marital status								
Unmarried			70	28.2				
Married			177	71.4				
Unknown			1	0.4				
Income								
$50,000 or less			40	16.1				
$50,000–$100,000			101	40.7				
$100,000–$150,000			77	31.0				
$150,000 or more			27	10.9				
Unknown			3	1.2				
Race and ethnicity								
NL White			223	89.9				
Black			3	1.2				
Latine			1	0.4				
Asian			4	1.6				
Multiracial			11	4.4				
Unknown			6	2.4				

*M*(*SD*) = mean (standard deviation); *Min* minimum, *Max* maximum, *NL White* Non-Latine White, *winsorized* winsorized to two standard deviations

^a^The Active Proportion when examining > 2 practices was 0.28, when examining > 3 practices was 0.28 and when examining > 9 practices was 0.29. When examining just the first 7 days of engagement for individuals who engaged in > 1 practice, Active Proportion was 0.30

^b^ T1 Psychological Distress at baseline (*n* = 246)

^c^T2 Psychological Distress at post-treatment (*n* = 228)

^d^T3 Psychological Distress at 3-month follow-up *(n* = 228)

### Sample 1

For the first research question (how much sitting or active meditation is used when both are offered), we utilized paired sample *t*-tests. Individuals engaged in more sitting practice (*M* = 5.88 practices, *SD* = 7.68) relative to active practice (*M* = 3.39 practices, *SD* = 6.31), *t*(26,531) = 43.26, *p* < 0.001, Cohen’s *d* = 0.35, 95% CI [0.33, 0.37].

For the second research question (demographic correlates of sitting versus active meditation), we used linear regression with Active Proportion as the outcome and demographic variables as the predictors (Table 4). There was a significant positive association between Active Proportion and identifying as a woman (*β* = 0.06, 95% CI [0.05, 0.07], *t*(26,516) = 9.50, *p* < 0.001), or other gender (*β* = 0.02, 95% CI [0.01, 0.03], *t*(26,516) = 2.84, *p* = 0.004) relative to identifying as a man. No other demographic associations were significant.

**Table 4:** Predicting active proportion by demographics for Sample 1

Demographic variable	Observation period	Number of practice sessions	*β*	95% CI	*p*
Woman	30	> 1	0.06	[0.05, 0.07]	< 0.001
Other gender	30	> 1	0.02	[0.01, 0.03]	0.004
Gender unknown	30	> 1	0.01	[− 0.00, 0.02]	0.119
Age 34 or less	30	> 1	0.00	[− 0.01, 0.02]	0.642
Age unknown	30	> 1	− 0.01	[− 0.02, 0.00]	0.164
College grad or higher	30	> 1	0.00	[− 0.01, 0.01]	0.960
Education unknown	30	> 1	0.00	[− 0.02, 0.02]	0.820
Married or domestic partnership	30	> 1	0.01	[− 0.00, 0.02]	0.106
Marital status unknown	30	> 1	0.00	[− 0.02, 0.01]	0.655
African American	30	> 1	0.00	[− 0.01, 0.01]	0.884
Latine	30	> 1	0.00	[− 0.01, 0.01]	0.689
Asian	30	> 1	0.00	[− 0.01, 0.02]	0.640
Native American or Pacific Islander	30	> 1	0.01	[− 0.00, 0.02]	0.112
Other	30	> 1	0.01	[− 0.00, 0.02]	0.105
Race unknown	30	> 1	0.01	[− 0.01, 0.03]	0.202

Active Proportion = the proportion of active meditation practice divided by the overall number of practices. *β* = standardized coefficient. The sample size was 26,532. The observation period was 30 days and only participants who engaged in more than one session were included. We utilize a Bonferroni correction and only interpret results where the *p*-value is less than 0.01

Sensitivity analyses assessed if the results changed when limiting the sample to varying numbers of minimum practice sessions, changing our observation period from 30 to 7 days, or covarying Sum Days (Supplemental Tables 4 and 5). The association between Active Proportion and identifying as a woman or other gender was significant across all sensitivity analyses except when the observation period was narrowed to 7 days. Additionally, the association between individuals identifying as other gender was non-significant when practice sessions were limited to at least 10 sessions.

For the third research question (whether utilization varies based on the proportion of active meditation), we used Sum Practice (winsorized) and Sum Days as outcomes and Active Proportion (linear and quadratic terms) as predictors (Table 5). We included demographic covariates in all models. We found a negative association between Sum Practice (winsorized) and the linear Active Proportion term (*β* =  − 0.62, 95% CI [− 0.66, − 0.58], *t*(26,514) =  − 31.99, *p* < 0.001) and a positive association between Sum Practice and the quadratic Active Proportion term (*β* = 0.55, 95% CI [0.51, 0.59], *t*(26,514) = 28.64, *p* < 0.001), indicating a non-linear relationship. Model-based predicted values showed that individuals who engaged in mostly sitting practice (active proportion = 0.05; *M* = 9.39 practices, 95% CI [9.08, 9.70]) or mostly active practice (active proportion = 0.95; *M* = 8.74 practices, 95% CI [8.38, 9.10]) were predicted to engage in more total practice than those who evenly used sitting and active practices (active proportion = 0.50; *M* = 6.47 practices, 95% CI [6.17, 6.76]; see Supplemental Figs. 1). The results were consistent for the association between Sum Days and the linear (*β* =  − 0.60, 95% CI [− 0.64, − 0.56], *t*(26,514) =  − 31.30, *p* < 0.001) and quadratic (*β* = 0.50, 95% CI [0.47, 0.54], *t*(26,514) = 26.25,* p* < 0.001) Active Proportion terms. Model-based predicted values similarly showed that individuals who engaged in mostly sitting practice (active proportion = 0.05; *M* = 8.57 practices, 95% CI [8.32, 8.81]) or mostly active practice (active proportion = 0.95; *M* = 7.29 practices, 95% CI [7.00, 7.57]) were predicted to engage in more total practice than those who evenly used sitting and active practices (active proportion = 0.50; *M* = 6.04 practices, 95% CI [5.80, 6.28]; see Supplemental Fig. 2). The results were also consistent across all sensitivity analyses including when we varied the minimum number of practice sessions, limited the observation period to 7 days, or used the raw rather than winsorized version of Sum Practice (Supplemental Table 6).

**Table 5:** Predicting utilization outcomes from active proportion in Sample 1

Outcome variable	Active proportion variable	Observation period	Number of practice sessions	*β*	95% CI	*p*
Sum Practice (winsorized)	Active Proportion	30	> 1	− 0.62	[− 0.66, − 0.58]	< 0.001
Sum Practice (winsorized)	Active Proportion^2^	30	> 1	0.55	[0.51, 0.59]	< 0.001
Sum Days	Active Proportion	30	> 1	− 0.60	[− 0.64, − 0.56]	< 0.001
Sum Days	Active Proportion^2^	30	> 1	0.50	[0.47, 0.54]	< 0.001

Active Proportion = the proportion of active meditation practice divided by the overall number of practices. *β* = standardized coefficient. The sample size 26,532. Demographics were included as covariates in all models

### Sample 2

For the first research question (how much sitting or active meditation is used when both are offered), we again observed that individuals engaged in more sitting practice (*M* = 13.68 practices, *SD* = 7.89) relative to active practice (*M* = 5.67 practices, *SD* = 6.17), *t*(247) = 11.23, *p* < 0.001, *d* = 1.13, 95% CI [0.89, 1.39]).

For the second research question (demographic and psychological distress correlates of sitting versus active meditation), we used linear regression with Active Proportion as the outcome and demographic variables and T1 Psychological Distress as the predictors (Table 6). There was a significant positive association between Active Proportion and individuals whose race was unknown (*β* = 0.17, 95% CI [0.04, 0.29], *t*(231) = 2.57, *p* = 0.011). Sensitivity analyses assessed if the results changed when limiting the sample to varying numbers of minimum practice sessions, changing our observation period from 30 to 7 days, or covarying Sum Days. The association between Active Proportion and race unknown was significant across all sensitivity analyses except when the observation period was narrowed to 7 days (Supplemental Tables 7 and 8).

**Table 6:** Predicting active proportion by demographics for Sample 2

Demographic variable	Number of days of app engagement	Number of practice sessions	*β*	95% CI	*p*
Woman	30	> 1	0.05	[− 0.08, 0.18]	0.460
Gender unknown	30	> 1	− 0.05	[− 0.18, 0.08]	0.455
Age	30	> 1	− 0.06	[− 0.20, 0.07]	0.340
College grad or higher	30	> 1	− 0.01	[− 0.14, 0.13]	0.912
Education unknown	30	> 1	− 0.07	[− 0.20, 0.05]	0.259
Married or domestic partnership	30	> 1	0.05	[− 0.11, 0.22]	0.511
Marital status unknown	30	> 1	− 0.03	[− 0.16, 0.10]	0.617
$50,000–$100,000	30	> 1	0.17	[− 0.04, 0.38]	0.121
$100,000–$150,000	30	> 1	0.16	[− 0.07, 0.40]	0.169
$150,000 or more	30	> 1	0.02	[− 0.17, 0.21]	0.844
Income unknown	30	> 1	0.09	[− 0.04, 0.23]	0.173
Race other	30	> 1	0.03	[− 0.10, 0.16]	0.679
Race unknown	30	> 1	0.17	[0.04, 0.29]	0.011
T1 distress	30	> 1	− 0.08	[− 0.21, 0.06]	0.259

Active Proportion = the proportion of active meditation practice divided by the overall number of practices. *β* = standardized coefficient. For 30 days of engagement, the sample size when examining > 1 practices was 248. A Bonferroni correction was applied such that only *p*-values which were smaller than 0.007 were interpreted

For the third research question (whether utilization or psychological outcomes vary based on the proportion of active meditation), we used Sum Practice, Sum Days, T2 Psychological Distress and T3 Psychological Distress as outcomes, and Active Proportion (linear and quadratic terms) as predictors (Table 7). We again included demographic covariates in all models. Patterns of finding in Sample 2 were contrary to Sample 1. In Sample 2, we observed a positive association between Sum Practice and the linear Active Proportion term (*β* = 0.57, 95% CI [0.16, 0.98], *t*(229) = 2.75, *p* = 0.007) and a negative association between Sum Practice and the quadratic Active Proportion term (*β* =  − 0.43, 95% CI [− 0.84, − 0.02], *t*(229) =  − 2.08, *p* = 0.039), indicating a non-linear relationship. Model-based predicted values showed that individuals who engaged in a combination of sitting and active practices (active proportion = 0.50; *M* = 23.60 practices, 95% CI [12.25, 34.95]) were predicted to engage in more total practice than those who primarily engaged in sitting practice (active proportion = 0.05; *M* = 19.49 practices, 95% CI [8.45, 30.54]) or mostly active practice (active proportion = 0.95; *M* = 19.87 practices, 95% CI [7.85, 31.88]; see Supplemental Fig. 3). The results were consistent for the association between Sum Days and the linear (*β* = 0.54, 95% CI [0.14, 0.95], *t*(229) = 2.68, *p* = 0.008) and quadratic (*β* =  − 0.43, 95% CI [− 0.83, − 0.03], *t*(229) =  − 2.11, *p* = 0.036) Active Proportion terms. Model-based predicted values similarly showed that individuals who engaged in a combination of sitting and active practices (active proportion = 0.50; *M* = 15.87 practices, 95% CI [6.34, 25.41]) were predicted to engage in more total practice days than those who primarily engaged in sitting practice (active proportion = 0.05; *M* = 12.71 practices, 95% CI [3.43, 21.99]) or mostly active practice (active proportion = 0.95; *M* = 12.43 practices, 95% CI [2.34, 22.53]; see Supplemental Fig. 4). The results were also consistently significant across all sensitivity analyses for both outcomes except when the number of practices were limited to 10 or more and when observation period was narrowed to 7 days (Supplemental Table 9).

**Table 7:** Predicting utilization and clinical outcomes from active proportion in Sample 2

Outcome variable	Active proportion variable	Number of days of app engagement	Number of practices	*β*	95% CI	*p*
Sum Practice	Active Proportion	30	> 1	0.57	[0.16, 0.98]	0.007
Sum Practice	Active Proportion^2^	30	> 1	− 0.43	[− 0.84, − 0.02]	0.039
Sum Days	Active Proportion	30	> 1	0.54	[0.14, 0.95]	0.008
Sum Days	Active Proportion^2^	30	> 1	− 0.43	[− 0.83, − 0.03]	0.036
T2 Psychological Distress	Active Proportion	30	> 1	0.35	[0.01, 0.69]	0.043
T2 Psychological Distress	Active Proportion^2^	30	> 1	− 0.30	[− 0.64, 0.04]	0.083
T3 Psychological Distress	Active Proportion	30	> 1	0.35	[0.03, 0.67]	0.034
T3 Psychological Distress	Active Proportion^2^	30	> 1	− 0.40	[− 0.72, − 0.08]	0.016

Active Proportion = the proportion of active meditation practice divided by the overall number of practices. *β* = standardized coefficient. The sample size for the analyses was 248. Demographics and T1 Psychological Distress were included as covariates in all models

With T2 Psychological Distress as the outcome (Table 7), we observed a significant positive association with the linear Active Proportion (*β* = 0.35, 95% CI [0.01, 0.69], *t*(210) = 2.03, *p* = 0.043) term, but not the quadratic (*β* =  − 0.30, 95% CI [− 0.64, 0.04], *t*(210) =  − 1.74, *p* = 0.083) term in our primary analysis. However, for T3 Psychological Distress, we observed a positive association with the linear Active Proportion term (*β* = 0.35, 95% CI [0.03, 0.67], *t*(210) = 2.14, *p* = 0.034) and a negative association with the quadratic Active Proportion term (*β* =  − 0.40, 95% CI [− 0.72, − 0.08], *t*(210) =  − 2.43, *p* = 0.016), indicating a non-linear relationship. Model-based predicted values showed that individuals who engaged in mostly sitting practice (active proportion = 0.05; *M* =  − 0.89 practices, 95% CI [− 1.58, − 0.20]) or mostly active practice (active proportion = 0.95; *M* =  − 1.31 practices, 95% CI [− 2.09, − 0.53]) were predicted to experience greater reductions in psychological distress relative to those who evenly used sitting and active practices (active proportion = 0.50; *M* =  − 0.78 practices, 95% CI [− 1.49, − 0.07]; see Supplemental Fig. 6). For T2 Psychological Distress, the results were consistent across all sensitivity analyses except when the number of practices was limited to 10 or more where the quadratic Active Proportion term was significant (*β* =  − 0.43, 95% CI [− 0.82, − 0.04], *t*(181) =  − 2.15, *p* = 0.033) and when observation period was narrowed to 7 days when the linear term was non-significant. For T3 Psychological Distress, the findings from the main model were consistent across all sensitivity analyses except when the observation period was narrowed to 7 days in which case it was non-significant (Supplemental Table 9).

In sensitivity analyses, we disaggregated psychological distress and examined depression, anxiety, and stress separately. The results differed by outcome and time point. At T2, we observed positive associations between linear Active Proportion and depression and anxiety, but not stress, as well as a negative association between quadratic Active Proportion and anxiety. At T3, positive associations with linear Active Proportion were again observed for depression and anxiety, but not stress, along with negative associations with quadratic Active Proportion for depression and anxiety, but not stress (Supplemental Table 10).

## Discussion

The current study explored the use of active meditation practices that involve periods of formal meditation while completing daily activities such as folding laundry or doing the dishes. Using data from two distinct contexts—public users of the Healthy Minds Program (HMP) app (*N* = 26,532, Sample 1) and the HMP arm of a recently completed randomized controlled trial (*N* = 248, Sample 2)—we examine the use of active practices and their associations with demographic characteristics, psychological distress, app utilization, and outcome variables. In both samples, we found evidence that participants more often chose sitting practices, although a sizable proportion of practices completed were active practices (38% and 28%, in Samples 1 and 2, respectively). This suggests that active practices are indeed perceived as engaging by a substantial portion of those interacting with HMP.

We found limited evidence that engagement with active practices varied across demographic groups. Within public users of the HMP app (Sample 1), women and those identifying as other gender had a higher proportion of active practices, though associated effect sizes were very small (*β* ≤ 0.06). Gender was not associated with proportion of active practices in the randomized controlled trial data (Sample 2), although this sample was predominantly women. Socioeconomic indicators (education and income) were not associated with proportion of active practices in either sample, contrary to the theoretical possibility that active practices may make meditation practice more accessible for lower socioeconomic status individuals. Similarly, race/ethnicity was generally not associated with proportion of active practices with the exception of race unknown in Sample 2. Participants’ baseline distress was also not associated with proportion of active practices. Results were generally consistent across sensitivity analyses except in some instances when we narrowed the observation to 7 days, instead of 30 days (Supplemental Tables 4 and 7). Taken together, this suggests that participants tend to use active practices at a similar rate regardless of baseline demographic characteristics and level of psychological distress.

Proportion of active practices was linked to patterns of app engagement in both samples. However, the specific patterns were directly opposite across the two samples (although no direct statistical comparison between the two was conducted). Among public users of the HMP, those who stuck with one or the other style of practice (i.e., predominantly sitting or active) tended to show higher rates of HMP utilization. Within the trial data, the exact opposite pattern was found (although again, the samples were not directly compared statistically)—participants who tended to use a combination of sitting and active practices showed higher rates of HMP utilization. Results from the trial data were also less consistent across sensitivity analyses, which may be expected given the smaller sample size relative to the public users.

The inconsistency highlighted across samples underscores the possibility that conclusions drawn from tightly controlled clinical trial contexts may not generalize to the broader population (Weiss et al., 2008). This highlights the value of pragmatic trials that seek to mirror real-world contexts as much as possible (MacPherson, 2004). Given the vastly larger sample size in the public users of the HMP, it seems most likely that adopting primarily a sitting or active practice habit supports greater utilization. Although this should be tested in future studies, this possibility is consistent with the habit formation literature where a single style of practice may promote automaticity and ultimately greater consistency (Lally et al., 2010). Selecting the same style of practice and not moving between the two styles may have reduced the cognitive load associated with completing a practice (Muraven & Baumeister, 2000), thereby also promoting engagement. This possibility as well will need to be studied directly in the future. Coupled with the large proportion of the sample who engaged with active practices, this is consistent with the notion that active practices are, for some individuals, an attractive form of meditation practice that does not diminish and may even increase one’s ability to persist with practice. Of course, given the correlational nature of the data, it is not possible to determine whether active practices cause increases in persistence. Results examining linkages between proportion of active practices and changes in psychological distress further support the viability of active practices. Although results were somewhat inconsistent across sensitivity analyses (i.e., when restricting the sample to participants engaging in 10 or more practices or when narrowing the observation window to 7 days; see Supplemental Table 9), the general pattern of findings suggests that those engaged with primarily sitting and perhaps even more so those engaged primarily in active practices (Supplemental Fig. 5 and 6)—rather than a combination of sitting and active practices—showed the largest decreases in psychological distress at long-term follow-up. At the very least, these results indicate that active practices appear no less effective than sitting practice. This is consistent with several randomized trials that have examined informal practice (e.g., Hanley et al., 2015; Shankland et al., 2021), including studies that have demonstrated informal practice may be similarly or more effective than formal practice when compared directly (e.g., Petrovic et al., 2025).

### Limitations and Future Research

This study has several important limitations. In regard to generalizability, both samples were predominantly women and NL White individuals, many individuals were excluded from the public use data either for not completing two practices or for not reporting any demographic data (Sample 1), and the randomized controlled trial data (Sample 2) occurred in a very specific context (school employees in Wisconsin), which all limit generalizability of study results to other groups. In regard to statistical power, although some analyses involving demographic variables may have been adequately powered (e.g., associations with demographic characteristics in Sample 1), low power (i.e., type II error) is a potential limitation for other key analyses (e.g., the degree to which proportion of active practices are associated with outcomes as this was only assessed in Sample 2). Further, *p*-value adjustments were not applied to all models, which may have inflated risk of type I error. It will be important to continue exploring active practices within large and diverse samples. It would be worthwhile conducting a study specifically focusing on the degree to which active practices offer a more acceptable form of guided meditation practice for groups who may be less likely to engage with and persist with meditation (e.g., non-college educated, African American, men; Jiwani et al., 2023; Macinko & Upchurch, 2019; Olano et al., 2015). Indeed, there are a wide variety of candidate characteristics that may predict who is most likely to gravitate towards and/or benefit from active practices (e.g., extraversion, restlessness, motivation) that could be used to guide practice recommendations. In addition, there are host of contextual factors that may also have influenced the patterns of findings (e.g., COVID-19 pandemic for Sample 2).

Another key methodological limitation noted previously, all associations between the proportion of active practices and other study variables are purely correlational. Thus, no causal claims can be made regarding the impact of engaging in active practices. The intriguing associations between active practices and outcomes in the current study suggest they may be ripe for further exploration using experimental paradigms. An obvious next study would be comparing the effects of a meditation intervention like HMP delivered exclusively through active practices versus a version delivered exclusively through sitting practices. Given the possibility that the effects of active or sitting practices is more momentary in nature, a micro-randomized trial design (Klasnja et al., 2015) would also be a valuable approach. A study could repeatedly randomize the assignment of practice posture (i.e., sitting versus active) each time a participant is engaging in a guided meditation practice. By assessing proximal outcomes after a given practice (e.g., mood immediately following the practice session or at the end of the day), one could evaluate the short-term impact of active versus sitting practices. Both a traditional randomized trial design as well as a micro-randomized trial design could be used to identify baseline (or in the case of a micro-randomized trial, momentary) characteristics that predict response to active versus sitting practices. These results could inform the personalization of meditation training in keeping with the spirit of personalized medicine (Hamburg & Collins, 2010; Nahum-Shani et al., 2017; Webb et al., 2022). A future version of the HMP or other meditation apps might use passively acquired data (e.g., GPS, physiological data from wearable sensors) that contain signals that could guide the delivery of sitting versus active practices.

One other methodological limitation is that data collected in both samples did not capture participants’ experience using sitting or active practice nor the reasons why participants may have chosen to engage in one or the other practice type. This also limits inferences we can make about how participants used each type of practice (e.g., during what activities did participants complete active practices). It would be valuable to conduct future, ideally qualitative, studies in order to understand these dynamics as well as to clarify who may be most likely to benefit from sitting and/or active practices. It would be very helpful to understand the specific activities that participants completed while doing active practices, given these activities and their associated features (e.g., cognitive load, physicality) may strongly impact the effects of active practices. This information was unfortunately not available in the current samples. It may also be helpful to assess the variability in motivations of meditators choosing sitting versus active practice to better understand the underlying individual differences that guide practice preferences (Jiwani et al., 2022). It would also be valuable for future studies to assess additional aspects of the practice itself. Although we gathered utilization data objectively through HMP, it would be helpful for future studies to evaluate the quality of practice (Del Re et al., 2013; Goldberg et al., 2020a, 2020b) along with quantity. A variety of other variables (e.g., affect during practice, time of day, stress level; Berardi et al., 2023; Goldberg et al., 2024) may impact the effect of active practices and are important to evaluate in future studies.

A final key future direction is further theoretical work examining the distinctions and potential overlap between formal and informal meditation practice, and where practices such as active meditation may fit within this scheme (or continuum). The fact that active practices were used a substantial amount of the time (28–38% of practices) and that the use of active practices was not associated with poorer clinical effects supports ongoing work studying this form of practice. Ritual theory (Bell, 1991) may contribute significantly to formulating a framework for characterizing the defining features of these categories which can guide further refinement of our understanding of this form of practice. In particular, it may be helpful to clarify when a mental behavior qualifies as an element that lacks direct instrumental purpose and therefore may meet the definition of ritual offered by Hobson et al. (2018), even when the practice is done in combination with elements that have instrumental purpose, such as folding laundry.

This study explores active practices as a form of meditation practice that, in theory, may increase the accessibility of formal meditation practice and support the integration of practice within daily life. Results indicated that when active practices are presented as a viable form of meditation practice, they are used fairly frequently and at generally consistent rates across demographic groups. The degree to which the proportion of active practices is associated with overall utilization of a meditation app varied between public users of the HMP app and data from a clinical trial. Primarily using active practices was not associated with poorer clinical outcomes and may even result in larger benefits relative to those who use a mixture of active and sitting practices. In summary, active practices appear to be a potentially attractive alternative to sitting practices that are not associated with poorer clinical effects. Thus, active practice appears worthy of further investigation using experimental methodologies (i.e., randomization).

## Supplementary Information

Below is the link to the electronic supplementary material.

ESM 1 (DOCX 99.5 KB)

## Data Availability

Code for Sample 1 along with the code and data for Sample 2 are available on the Open Science Framework ([https://osf.io/8pzvr/?view_only=18fe4db8e5d54845b5868c91bcc31fc6]).

## Declarations

### Use of Artificial Intelligence

Artificial intelligence (AI) was used for assisting with conducting literature reviews and for creating statistical analysis code.
