# Supplementary material for: Sitting and Active Meditation Practice: Utilization and Associations with Outcomes in Naturalistic and Clinical Trial Data
Source: Mindfulness (N Y). 2026 Mar 9;17(4):1120–37. doi: 10.1007/s12671-026-02787-w (PMC12971069; doi:10.1007/s12671-026-02787-w)
Supplement: Supplementary file 1 — (DOCX 99.5 KB) [file 12671_2026_2787_MOESM1_ESM.docx]

**Supplemental Table 1**

Predicting Utilization Outcomes from Active Proportion in Sample 1 with Linear, Quadratic and Cubic Terms Entered Simultaneously

| **Outcome Variable** | **Active Proportion Variable** | **Observation Period** | **Number of Practice Sessions** | ***β*** | **95% CI** | ***p*** |
| --- | --- | --- | --- | --- | --- | --- |
| Sum Practice | Active Proportion | 30 | >1 | 0.04 | [-0.05, 0.12] | 0.420 |
| Sum Practice | Active Proportion^2^ | 30 | >1 | -0.94 | [-1.15, -0.73] | <0.001 |
| Sum Practice | Active Proportion^3^ | 30 | >1 | 0.93 | [0.80, 1.07] | <0.001 |
| Sum Practice | Active Proportion | 30 | >2 | -0.41 | [-0.51, -0.31] | <0.001 |
| Sum Practice | Active Proportion^2^ | 30 | >2 | 0.07 | [-0.17, 0.31] | 0.567 |
| Sum Practice | Active Proportion^3^ | 30 | >2 | 0.36 | [0.20, 0.51] | <0.001 |
| Sum Practice | Active Proportion | 30 | >3 | -0.42 | [-0.54, -0.30] | <0.001 |
| Sum Practice | Active Proportion^2^ | 30 | >3 | 0.26 | [-0.02, 0.55] | 0.071 |
| Sum Practice | Active Proportion^3^ | 30 | >3 | 0.19 | [0.01, 0.37] | 0.043 |
| Sum Practice | Active Proportion | 30 | >9 | -0.01 | [-0.22, 0.19] | 0.887 |
| Sum Practice | Active Proportion^2^ | 30 | >9 | -0.21 | [-0.71, 0.28] | 0.396 |
| Sum Practice | Active Proportion^3^ | 30 | >9 | 0.31 | [-0.01, 0.62] | 0.058 |
| Sum Practice | Active Proportion | 7 | >1 | 0.35 | [0.27, 0.44] | <0.001 |
| Sum Practice | Active Proportion^2^ | 7 | >1 | -1.53 | [-1.73, -1.32] | <0.001 |
| Sum Practice | Active Proportion^3^ | 7 | >1 | 1.24 | [1.10, 1.38] | <0.001 |
| Sum Practice (winsorized) | Active Proportion | 30 | >1 | 0.09 | [0.00, 0.17] | 0.046 |
| Sum Practice (winsorized) | Active Proportion^2^ | 30 | >1 | -1.33 | [-1.53, -1.12] | <0.001 |
| Sum Practice (winsorized) | Active Proportion^3^ | 30 | >1 | 1.24 | [1.10, 1.37] | <0.001 |
| Sum Practice (winsorized) | Active Proportion | 30 | >2 | -0.63 | [-0.73, -0.54] | <0.001 |
| Sum Practice (winsorized) | Active Proportion^2^ | 30 | >2 | 0.32 | [0.08, 0.56] | 0.008 |
| Sum Practice (winsorized) | Active Proportion^3^ | 30 | >2 | 0.28 | [0.13, 0.43] | <0.001 |
| Sum Practice (winsorized) | Active Proportion | 30 | >3 | -0.73 | [-0.85, -0.61] | <0.001 |
| Sum Practice (winsorized) | Active Proportion^2^ | 30 | >3 | 0.76 | [0.48, 1.05] | <0.001 |
| Sum Practice (winsorized) | Active Proportion^3^ | 30 | >3 | -0.06 | [-0.24, 0.12] | 0.541 |
| Sum Practice (winsorized) | Active Proportion | 30 | >9 | -0.35 | [-0.55, -0.15] | <0.001 |
| Sum Practice (winsorized) | Active Proportion^2^ | 30 | >9 | 0.54 | [0.04, 1.04] | 0.034 |
| Sum Practice (winsorized) | Active Proportion^3^ | 30 | >9 | -0.18 | [-0.50, 0.14] | 0.259 |
| Sum Practice (winsorized) | Active Proportion | 7 | >1 | 0.63 | [0.55, 0.72] | <0.001 |
| Sum Practice (winsorized) | Active Proportion^2^ | 7 | >1 | -2.38 | [-2.59, -2.17] | <0.001 |
| Sum Practice (winsorized) | Active Proportion^3^ | 7 | >1 | 1.79 | [1.66, 1.93] | <0.001 |
| Sum Days | Active Proportion | 30 | >1 | -0.03 | [-0.12, 0.05] | 0.437 |
| Sum Days | Active Proportion^2^ | 30 | >1 | -1.01 | [-1.22, -0.81] | <0.001 |
| Sum Days | Active Proportion^3^ | 30 | >1 | 1.00 | [0.86, 1.13] | <0.001 |
| Sum Days | Active Proportion | 30 | >2 | -0.70 | [-0.80, -0.60] | <0.001 |
| Sum Days | Active Proportion^2^ | 30 | >2 | 0.52 | [0.28, 0.75] | <0.001 |
| Sum Days | Active Proportion^3^ | 30 | >2 | 0.10 | [-0.05, 0.25] | 0.182 |
| Sum Days | Active Proportion | 30 | >3 | -0.80 | [-0.92, -0.69] | <0.001 |
| Sum Days | Active Proportion^2^ | 30 | >3 | 0.94 | [0.66, 1.23] | <0.001 |
| Sum Days | Active Proportion^3^ | 30 | >3 | -0.22 | [-0.40, -0.04] | 0.019 |
| Sum Days | Active Proportion | 30 | >9 | -0.60 | [-0.80, -0.40] | <0.001 |
| Sum Days | Active Proportion^2^ | 30 | >9 | 0.94 | [0.45, 1.43] | <0.001 |
| Sum Days | Active Proportion^3^ | 30 | >9 | -0.43 | [-0.74, -0.11] | 0.008 |
| Sum Days | Active Proportion | 7 | >1 | 0.63 | [0.54, 0.71] | <0.001 |
| Sum Days | Active Proportion^2^ | 7 | >1 | -2.16 | [-2.36, -1.95] | <0.001 |
| Sum Days | Active Proportion^3^ | 7 | >1 | 1.51 | [1.37, 1.64] | <0.001 |

*Note.* Active Proportion = the proportion of active meditation practice divided by the overall number of practices. *β* = standardized coefficient. For 30 days of engagement, the sample size when examining >1 practices was 26,532. For >2 practices *n* = 21,675, >3 practices *n* = 18,014, >9 practices *n* = 8,345. When examining just the first seven days of engagement for individuals who engaged in >1 practice, *n* = 21,248. Demographics were included as covariates in all models.

**Supplemental Table 2**

Predicting Utilization and Clinical Outcomes from Active Proportion in Sample 2 with Linear, Quadratic and Cubic Terms Entered Simultaneously

| **Outcome Variable** | **Active Proportion Variable** | **Number of Days of App Engagement** | **Number of Practices** | ***β*** | ***9*5%CI** | ***p*** |
| --- | --- | --- | --- | --- | --- | --- |
| Sum Practice | Active Proportion | 30 | >1 | 1.30 | [0.40, 2.20] | 0.005 |
| Sum Practice | Active Proportion^2^ | 30 | >1 | -2.31 | [-4.43, -0.19] | 0.033 |
| Sum Practice | Active Proportion^3^ | 30 | >1 | 1.21 | [-0.13, 2.55] | 0.076 |
| Sum Practice | Active Proportion | 30 | >2 | 1.04 | [0.05, 1.84] | 0.027 |
| Sum Practice | Active Proportion^2^ | 30 | >2 | -1.45 | [-3.55, 0.62] | 0.188 |
| Sum Practice | Active Proportion^3^ | 30 | >2 | 0.60 | [-0.66, 1.99] | 0.392 |
| Sum Practice | Active Proportion | 30 | >3 | 0.74 | [-0.72, 0.81] | 0.135 |
| Sum Practice | Active Proportion^2^ | 30 | >3 | -0.83 | [-1.32, 2.27] | 0.473 |
| Sum Practice | Active Proportion^3^ | 30 | >3 | 0.21 | [-1.63, 0.63] | 0.772 |
| Sum Practice | Active Proportion | 30 | >9 | 0.30 | [-0.43, 1.03] | 0.616 |
| Sum Practice | Active Proportion^2^ | 30 | >9 | -0.51 | [-1.98, 1.46] | 0.717 |
| Sum Practice | Active Proportion^3^ | 30 | >9 | 0.32 | [-1.18, 1.00] | 0.716 |
| Sum Practice | Active Proportion | 7 | >1 | 1.11 | [0.12, 1.96] | 0.007 |
| Sum Practice | Active Proportion^2^ | 7 | >1 | -2.50 | [-3.62, 0.71] | 0.011 |
| Sum Practice | Active Proportion^3^ | 7 | >1 | 1.49 | [-0.78, 1.98] | 0.022 |
| Sum Days | Active Proportion | 30 | >1 | 0.95 | [-0.20, 1.61] | 0.038 |
| Sum Days | Active Proportion^2^ | 30 | >1 | -1.47 | [-2.83, 1.45] | 0.169 |
| Sum Days | Active Proportion^3^ | 30 | >1 | 0.67 | [-1.24, 1.47] | 0.321 |
| Sum Days | Active Proportion | 30 | >2 | 0.71 | [-0.69, 0.89] | 0.125 |
| Sum Days | Active Proportion^2^ | 30 | >2 | -0.69 | [-1.46, 2.24] | 0.526 |
| Sum Days | Active Proportion^3^ | 30 | >2 | 0.11 | [-1.63, 0.71] | 0.869 |
| Sum Days | Active Proportion | 30 | >3 | 0.45 | [-0.42, 1.06] | 0.356 |
| Sum Days | Active Proportion^2^ | 30 | >3 | -0.15 | [-2.02, 1.48] | 0.894 |
| Sum Days | Active Proportion^3^ | 30 | >3 | -0.22 | [-1.21, 1.02] | 0.758 |
| Sum Days | Active Proportion | 30 | >9 | -0.15 | [-0.23, 1.71] | 0.787 |
| Sum Days | Active Proportion^2^ | 30 | >9 | 0.82 | [-3.10, 1.44] | 0.545 |
| Sum Days | Active Proportion^3^ | 30 | >9 | -0.64 | [-1.22, 1.65] | 0.460 |
| Sum Days | Active Proportion | 7 | >1 | 0.42 | [-0.51, 1.40] | 0.286 |
| Sum Days | Active Proportion^2^ | 7 | >1 | -1.39 | [-2.39, 2.08] | 0.145 |
| Sum Days | Active Proportion^3^ | 7 | >1 | 0.95 | [-1.63, 1.19] | 0.130 |
| T2 Psychological Distress | Active Proportion | 30 | >1 | 0.05 | [-0.73, 0.87] | 0.906 |
| T2 Psychological Distress | Active Proportion^2^ | 30 | >1 | 0.48 | [-1.44, 2.32] | 0.602 |
| T2 Psychological Distress | Active Proportion^3^ | 30 | >1 | -0.50 | [-1.67, 0.71] | 0.387 |
| T2 Psychological Distress | Active Proportion | 30 | >2 | 0.10 | [-0.24, 1.28] | 0.801 |
| T2 Psychological Distress | Active Proportion^2^ | 30 | >2 | 0.39 | [-2.45, 1.13] | 0.676 |
| T2 Psychological Distress | Active Proportion^3^ | 30 | >2 | -0.46 | [-1.02, 1.25] | 0.437 |
| T2 Psychological Distress | Active Proportion | 30 | >3 | 0.07 | [-0.87, 1.46] | 0.866 |
| T2 Psychological Distress | Active Proportion^2^ | 30 | >3 | 0.44 | [-3.27, 2.26] | 0.647 |
| T2 Psychological Distress | Active Proportion^3^ | 30 | >3 | -0.48 | [-1.42, 2.07] | 0.425 |
| T2 Psychological Distress | Active Proportion | 30 | >9 | 0.26 | [-1.29, 0.98] | 0.590 |
| T2 Psychological Distress | Active Proportion^2^ | 30 | >9 | 0.14 | [-1.86, 3.51] | 0.900 |
| T2 Psychological Distress | Active Proportion^3^ | 30 | >9 | -0.37 | [-2.33, 1.06] | 0.607 |
| T2 Psychological Distress | Active Proportion | 7 | >1 | -0.15 | [-0.68, 1.19] | 0.658 |
| T2 Psychological Distress | Active Proportion^2^ | 7 | >1 | 0.45 | [-2.07, 2.36] | 0.583 |
| T2 Psychological Distress | Active Proportion^3^ | 7 | >1 | -0.30 | [-1.76, 1.03] | 0.585 |
| T3 Psychological Distress | Active Proportion | 30 | >1 | 0.30 | [0.07, 1.77] | 0.426 |
| T3 Psychological Distress | Active Proportion^2^ | 30 | >1 | -0.26 | [-3.48, 0.57] | 0.765 |
| T3 Psychological Distress | Active Proportion^3^ | 30 | >1 | -0.09 | [-0.72, 1.85] | 0.874 |
| T3 Psychological Distress | Active Proportion | 30 | >2 | 0.32 | [0.31, 1.90] | 0.394 |
| T3 Psychological Distress | Active Proportion^2^ | 30 | >2 | -0.27 | [-4.43, -0.57] | 0.758 |
| T3 Psychological Distress | Active Proportion^3^ | 30 | >2 | -0.09 | [0.21, 2.76] | 0.868 |
| T3 Psychological Distress | Active Proportion | 30 | >3 | 0.52 | [-0.35, 1.19] | 0.180 |
| T3 Psychological Distress | Active Proportion^2^ | 30 | >3 | -0.66 | [-3.25, 0.48] | 0.470 |
| T3 Psychological Distress | Active Proportion^3^ | 30 | >3 | 0.11 | [-0.28, 2.18] | 0.844 |
| T3 Psychological Distress | Active Proportion | 30 | >9 | 0.92 | [-0.83, 0.52] | 0.034 |
| T3 Psychological Distress | Active Proportion^2^ | 30 | >9 | -1.46 | [-1.17, 2.08] | 0.157 |
| T3 Psychological Distress | Active Proportion^3^ | 30 | >9 | 0.57 | [-1.36, 0.77] | 0.385 |
| T3 Psychological Distress | Active Proportion | 7 | >1 | 0.40 | [-0.23, 1.04] | 0.214 |
| T3 Psychological Distress | Active Proportion^2^ | 7 | >1 | -0.96 | [-2.50, 0.58] | 0.222 |
| T3 Psychological Distress | Active Proportion^3^ | 7 | >1 | 0.47 | [-0.55, 1.48] | 0.366 |

*Note.* Active Proportion = the proportion of active meditation practice divided by the overall number of practices. *β* = standardized coefficient. For 30 days of engagement, the sample size when examining >1 practices was 248. For >2 practices *n* = 240, >3 practices *n* = 231, >9 practices *n* = 207. When examining just the first seven days of engagement for individuals who engaged in >1 practice, *n* = 203. Demographics and T1 Psychological Distress were included as covariates in all models.

**Supplemental Table 3**

*Sample 2 Regression Assumption Diagnostics*

| **Outcome** | ***n*** | ***r*(fitted, resid)** | **Skewness** | **Excess kurtosis** | ***r*(\|resid\|, fitted)** | **BP *p*** | **Max adj. GVIF** |
| --- | --- | --- | --- | --- | --- | --- | --- |
| Active Proportion | 246 | 0.00 | 0.664 | -0.354 | 0.211 | 0.517 | 1.16 |
| Sum Practice | 246 | 0.00 | -0.457 | -0.745 | -0.086 | 0.782 | 3.26 |
| Sum Days | 246 | 0.00 | -0.122 | -0.823 | -0.012 | 0.733 | 3.26 |
| T2 Distress | 226 | 0.00 | -0.492 | 0.902 | 0.121 | 0.633 | 3.31 |
| T3 Distress | 226 | 0.00 | -0.244 | 1.162 | 0.11 | 0.35 | 3.28 |

*Note. n* indicates the analytic sample size. *r*(fitted, resid) is the correlation between fitted values and residuals serving as an index of linearity. Skewness and Excess kurtosis summarize the distribution of residuals. *r*(|resid|, fitted) is the correlation between the absolute residuals and fitted values and serves as a diagnostic of heteroscedasticity. BP *p* is the p-value from the Breusch–Pagan test of heteroscedasticity. Max adj. GVIF is the maximum adjusted generalized variance inflation factor across predictors, with lower values indicating minimal multicollinearity.

**Supplemental Table 4**

Sensitivity Analyses Predicting Active Proportion by Demographics for Sample 1

| **Demographic Variable** | **Observation Period** | **Number of Practice Sessions** | ***β*** | **95% CI** | ***p*** |
| --- | --- | --- | --- | --- | --- |
| Woman | 30 | >2 | 0.07 | [0.06, 0.08] | <0.001 |
| Woman | 30 | >3 | 0.08 | [0.06, 0.09] | <0.001 |
| Woman | 30 | >9 | 0.08 | [0.06, 0.10] | <0.001 |
| Woman | 7 | >1 | -0.01 | [-0.03, 0.00] | 0.102 |
| Other Gender | 30 | >2 | 0.02 | [0.01, 0.04] | 0.002 |
| Other Gender | 30 | >3 | 0.02 | [0.01, 0.04] | 0.001 |
| Other Gender | 30 | >9 | 0.02 | [-0.00, 0.04] | 0.072 |
| Other Gender | 7 | >1 | -0.01 | [-0.02, 0.01] | 0.266 |
| Gender Unknown | 30 | >2 | 0.02 | [0.00, 0.03] | 0.013 |
| Gender Unknown | 30 | >3 | 0.02 | [0.00, 0.04] | 0.023 |
| Gender Unknown | 30 | >9 | 0.02 | [-0.00, 0.05] | 0.052 |
| Gender Unknown | 7 | >1 | 0.00 | [-0.02, 0.01] | 0.529 |
| Age 34 or Less | 30 | >2 | 0.00 | [-0.01, 0.02] | 0.647 |
| Age 34 or Less | 30 | >3 | 0.01 | [-0.00, 0.03] | 0.108 |
| Age 34 or Less | 30 | >9 | 0.02 | [-0.01, 0.04] | 0.166 |
| Age 34 or Less | 7 | >1 | 0.00 | [-0.02, 0.01] | 0.528 |
| Age Unknown | 30 | >2 | -0.01 | [-0.03, 0.00] | 0.105 |
| Age Unknown | 30 | >3 | -0.01 | [-0.03, 0.00] | 0.144 |
| Age Unknown | 30 | >9 | -0.01 | [-0.03, 0.02] | 0.461 |
| Age Unknown | 7 | >1 | -0.01 | [-0.03, 0.00] | 0.058 |
| College Grad or Higher | 30 | >2 | 0.00 | [-0.02, 0.02] | 0.945 |
| College Grad or Higher | 30 | >3 | 0.01 | [-0.01, 0.02] | 0.532 |
| College Grad or Higher | 30 | >9 | 0.00 | [-0.03, 0.02] | 0.898 |
| College Grad or Higher | 7 | >1 | 0.01 | [-0.00, 0.03] | 0.134 |
| Education Unknown | 30 | >2 | -0.01 | [-0.03, 0.01] | 0.491 |
| Education Unknown | 30 | >3 | -0.01 | [-0.03, 0.02] | 0.508 |
| Education Unknown | 30 | >9 | -0.02 | [-0.05, 0.02] | 0.317 |
| Education Unknown | 7 | >1 | 0.02 | [0.00, 0.04] | 0.032 |
| Married or Domestic Partnership | 30 | >2 | 0.01 | [-0.00, 0.03] | 0.079 |
| Married or Domestic Partnership | 30 | >3 | 0.01 | [-0.00, 0.03] | 0.084 |
| Married or Domestic Partnership | 30 | >9 | 0.01 | [-0.02, 0.03] | 0.480 |
| Married or Domestic Partnership | 7 | >1 | 0.01 | [-0.00, 0.03] | 0.130 |
| Marital Status Unknown | 30 | >2 | 0.00 | [-0.02, 0.01] | 0.910 |
| Marital Status Unknown | 30 | >3 | 0.01 | [-0.01, 0.02] | 0.543 |
| Marital Status Unknown | 30 | >9 | -0.01 | [-0.03, 0.02] | 0.571 |
| Marital Status Unknown | 7 | >1 | 0.00 | [-0.02, 0.01] | 0.859 |
| African American | 30 | >2 | 0.01 | [-0.00, 0.03] | 0.077 |
| African American | 30 | >3 | 0.01 | [-0.00, 0.03] | 0.125 |
| African American | 30 | >9 | 0.02 | [0.00, 0.04] | 0.040 |
| African American | 7 | >1 | -0.01 | [-0.02, 0.01] | 0.386 |
| Latine | 30 | >2 | 0.00 | [-0.02, 0.01] | 0.826 |
| Latine | 30 | >3 | 0.00 | [-0.01, 0.02] | 0.557 |
| Latine | 30 | >9 | 0.00 | [-0.02, 0.03] | 0.668 |
| Latine | 7 | >1 | -0.01 | [-0.02, 0.01] | 0.342 |
| Asian | 30 | >2 | 0.01 | [-0.01, 0.02] | 0.335 |
| Asian | 30 | >3 | 0.00 | [-0.01, 0.02] | 0.676 |
| Asian | 30 | >9 | 0.02 | [-0.00, 0.04] | 0.086 |
| Asian | 7 | >1 | -0.01 | [-0.02, 0.00] | 0.182 |
| Native American or Pacific Islander | 30 | >2 | 0.01 | [-0.00, 0.02] | 0.102 |
| Native American or Pacific Islander | 30 | >3 | 0.00 | [-0.01, 0.02] | 0.688 |
| Native American or Pacific Islander | 30 | >9 | 0.01 | [-0.02, 0.03] | 0.592 |
| Native American or Pacific Islander | 7 | >1 | 0.02 | [0.00, 0.03] | 0.013 |
| Other | 30 | >2 | 0.01 | [-0.00, 0.02] | 0.097 |
| Other | 30 | >3 | 0.01 | [-0.00, 0.03] | 0.130 |
| Other | 30 | >9 | 0.02 | [-0.00, 0.04] | 0.077 |
| Other | 7 | >1 | 0.01 | [-0.01, 0.02] | 0.302 |
| Race Unknown | 30 | >2 | 0.01 | [-0.01, 0.03] | 0.369 |
| Race Unknown | 30 | >3 | 0.01 | [-0.01, 0.03] | 0.545 |
| Race Unknown | 30 | >9 | 0.00 | [-0.04, 0.03] | 0.808 |
| Race Unknown | 7 | >1 | 0.02 | [-0.00, 0.04] | 0.107 |

*Note.* Active Proportion = the proportion of active meditation practice divided by the overall number of practices. *β* = standardized coefficient. For 30 days of engagement, the sample size when examining >2 practices *n* = 21,675, >3 practices *n* = 18,014, >9 practices *n* = 8,345. When examining just the first seven days of engagement for individuals who engaged in >1 practice, *n* = 21,248. We utilize a Bonferroni correction and only interpret results where the p value is less than 0.01

**Supplemental Table 5**

Predicting Active Proportion by Demographics for Sample 1 Covarying Total Days of Engagement

| **Demographic Variable** | **Observation Period** | **Minimum Number of Sessions** | ***β*** | **95% CI** | ***p*** |
| --- | --- | --- | --- | --- | --- |
| Woman | 30 | >1 | 0.05 | [0.04, 0.06] | <0.001 |
| Other Gender | 30 | >1 | 0.02 | [0.00, 0.03] | 0.008 |
| Gender Unknown | 30 | >1 | 0.01 | [-0.00, 0.02] | 0.118 |
| Age 34 or Less | 30 | >1 | 0.00 | [-0.01, 0.01] | 0.846 |
| Age Unknown | 30 | >1 | -0.01 | [-0.02, 0.00] | 0.134 |
| College Grad or Higher | 30 | >1 | 0.02 | [0.00, 0.03] | 0.015 |
| Education Unknown | 30 | >1 | 0.01 | [-0.01, 0.03] | 0.426 |
| Married or Domestic Partnership | 30 | >1 | 0.01 | [0.00, 0.03] | 0.041 |
| Marital Status Unknown | 30 | >1 | 0.00 | [-0.02, 0.01] | 0.663 |
| African American | 30 | >1 | 0.00 | [-0.02, 0.01] | 0.554 |
| Latine | 30 | >1 | 0.00 | [-0.01, 0.01] | 0.891 |
| Asian | 30 | >1 | 0.00 | [-0.01, 0.01] | 0.682 |
| Native American or Pacific Islander | 30 | >1 | 0.01 | [-0.00, 0.02] | 0.221 |
| Other | 30 | >1 | 0.01 | [-0.00, 0.02] | 0.178 |
| Race Unknown | 30 | >1 | 0.01 | [-0.01, 0.03] | 0.302 |
| Sum Days | 30 | >1 | -0.12 | [-0.14, -0.11] | <0.001 |

*Note.* Active Proportion = the proportion of active meditation practice divided by the overall number of practices. *β* = standardized coefficient. The sample size was 26,532. The observation period was 30 days and only participants who engaged in more than one session were included.

**Supplemental Table 6**

Sensitivity Analyses Predicting Utilization Outcomes from Active Proportion in Sample 1

| **Outcome Variable** | **Active Proportion Variable** | **Observation Period** | **Number of Practice Sessions** | ***β*** | **95% CI** | ***p*** |
| --- | --- | --- | --- | --- | --- | --- |
| Sum Practice | Active Proportion | 30 | >1 | -0.50 | [-0.53, -0.46] | <0.001 |
| Sum Practice | Active Proportion^2^ | 30 | >1 | 0.48 | [0.44, 0.52] | <0.001 |
| Sum Practice | Active Proportion | 30 | >2 | -0.61 | [-0.66, -0.56] | <0.001 |
| Sum Practice | Active Proportion^2^ | 30 | >2 | 0.61 | [0.57, 0.66] | <0.001 |
| Sum Practice | Active Proportion | 30 | >3 | -0.53 | [-0.59, -0.48] | <0.001 |
| Sum Practice | Active Proportion^2^ | 30 | >3 | 0.55 | [0.50, 0.61] | <0.001 |
| Sum Practice | Active Proportion | 30 | >9 | -0.19 | [-0.27, -0.11] | <0.001 |
| Sum Practice | Active Proportion^2^ | 30 | >9 | 0.26 | [0.17, 0.34] | <0.001 |
| Sum Practice | Active Proportion | 7 | >1 | -0.35 | [-0.39, -0.31] | <0.001 |
| Sum Practice | Active Proportion^2^ | 7 | >1 | 0.35 | [0.31, 0.39] | <0.001 |
| Sum Practice (winsorized) | Active Proportion | 30 | >2 | -0.79 | [-0.84, -0.75] | <0.001 |
| Sum Practice (winsorized) | Active Proportion^2^ | 30 | >2 | 0.75 | [0.70, 0.80] | <0.001 |
| Sum Practice (winsorized) | Active Proportion | 30 | >3 | -0.69 | [-0.75, -0.64] | <0.001 |
| Sum Practice (winsorized) | Active Proportion^2^ | 30 | >3 | 0.67 | [0.62, 0.73] | <0.001 |
| Sum Practice (winsorized) | Active Proportion | 30 | >9 | -0.24 | [-0.33, -0.16] | <0.001 |
| Sum Practice (winsorized) | Active Proportion^2^ | 30 | >9 | 0.26 | [0.17, 0.34] | <0.001 |
| Sum Practice (winsorized) | Active Proportion | 7 | >1 | -0.38 | [-0.42, -0.34] | <0.001 |
| Sum Practice (winsorized) | Active Proportion^2^ | 7 | >1 | 0.33 | [0.29, 0.37] | <0.001 |
| Sum Days | Active Proportion | 30 | >2 | -0.76 | [-0.81, -0.71] | <0.001 |
| Sum Days | Active Proportion^2^ | 30 | >2 | 0.68 | [0.63, 0.72] | <0.001 |
| Sum Days | Active Proportion | 30 | >3 | -0.68 | [-0.73, -0.63] | <0.001 |
| Sum Days | Active Proportion^2^ | 30 | >3 | 0.61 | [0.56, 0.67] | <0.001 |
| Sum Days | Active Proportion | 30 | >9 | -0.36 | [-0.44, -0.27] | <0.001 |
| Sum Days | Active Proportion^2^ | 30 | >9 | 0.29 | [0.20, 0.37] | <0.001 |
| Sum Days | Active Proportion | 7 | >1 | -0.23 | [-0.27, -0.19] | <0.001 |
| Sum Days | Active Proportion^2^ | 7 | >1 | 0.12 | [0.08, 0.16] | <0.001 |

*Note.* Active Proportion = the proportion of active meditation practice divided by the overall number of practices. *β* = standardized coefficient. For 30 days of engagement, the sample size when examining >1 practice was 26,532, >2 practices *n* = 21,675, >3 practices *n* = 18,014, >9 practices *n* = 8,345. When examining just the first seven days of engagement for individuals who engaged in >1 practice, *n* = 21,248. Demographics were included as covariates in all models.

**Supplemental Table 7**

Sensitivity Analyses Predicting Active Proportion by Demographics for Sample 2

| **Demographic Variable** | **Number of Days of App Engagement** | **Number of Practice Sessions** | ***β*** | **95% CI** | ***p*** |
| --- | --- | --- | --- | --- | --- |
| Woman | 30 | >2 | 0.05 | [-0.08, 0.19] | .437 |
| Woman | 30 | >3 | 0.05 | [-0.08, 0.19] | .445 |
| Woman | 30 | >9 | 0.03 | [-0.11, 0.18] | .625 |
| Woman | 7 | >1 | 0.04 | [-0.11, 0.19] | .609 |
| Gender Unknown | 30 | >2 | -0.05 | [-0.18, 0.08] | .464 |
| Gender Unknown | 30 | >3 | -0.05 | [-0.19, 0.09] | .463 |
| Gender Unknown | 30 | >9 | -0.06 | [-0.20, 0.09] | .426 |
| Gender Unknown | 7 | >1 | -0.08 | [-0.23, 0.07] | .289 |
| Age | 30 | >2 | -0.07 | [-0.20, 0.07] | .325 |
| Age | 30 | >3 | -0.07 | [-0.21, 0.07] | .334 |
| Age | 30 | >9 | -0.06 | [-0.20, 0.09] | .44 |
| Age | 7 | >1 | 0.03 | [-0.13, 0.18] | .745 |
| College Grad or Higher | 30 | >2 | -0.01 | [-0.14, 0.13] | .938 |
| College Grad or Higher | 30 | >3 | 0.01 | [-0.13, 0.15] | .905 |
| College Grad or Higher | 30 | >9 | 0.00 | [-0.15, 0.14] | .996 |
| College Grad or Higher | 7 | >1 | 0.00 | [-0.15, 0.15] | .979 |
| Education Unknown | 30 | >2 | -0.07 | [-0.20, 0.06] | .272 |
| Education Unknown | 30 | >3 | -0.07 | [-0.21, 0.06] | .287 |
| Education Unknown | 30 | >9 | -0.10 | [-0.24, 0.04] | .180 |
| Education Unknown | 7 | >1 | -0.08 | [-0.23, 0.07] | .286 |
| Married or Domestic Partnership | 30 | >2 | 0.06 | [-0.10, 0.23] | .432 |
| Married or Domestic Partnership | 30 | >3 | 0.08 | [-0.09, 0.24] | .353 |
| Married or Domestic Partnership | 30 | >9 | 0.04 | [-0.14, 0.22] | .651 |
| Married or Domestic Partnership | 7 | >1 | 0.16 | [-0.03, 0.35] | .101 |
| Marital Status Unknown | 30 | >2 | -0.03 | [-0.16, 0.10] | 0.655 |
| Marital Status Unknown | 30 | >3 | -0.03 | [-0.16, 0.11] | 0.676 |
| $50,000-$100,000 | 30 | >2 | 0.17 | [-0.04, 0.38] | 0.115 |
| $50,000-$100,000 | 30 | >3 | 0.16 | [-0.06, 0.38] | 0.145 |
| $50,000-$100,000 | 30 | >9 | 0.19 | [-0.04, 0.41] | 0.104 |
| $50,000-$100,000 | 7 | >1 | -0.01 | [-0.25, 0.23] | 0.936 |
| $100,000-$150,000 | 30 | >2 | 0.19 | [-0.04, 0.43] | 0.107 |
| $100,000-$150,000 | 30 | >3 | 0.18 | [-0.06, 0.42] | 0.133 |
| $100,000-$150,000 | 30 | >9 | 0.21 | [-0.04, 0.46] | 0.094 |
| $100,000-$150,000 | 7 | >1 | -0.08 | [-0.34, 0.19] | 0.562 |
| $150,000 or more | 30 | >2 | 0.02 | [-0.17, 0.21] | 0.845 |
| $150,000 or more | 30 | >3 | 0.01 | [-0.18, 0.20] | 0.901 |
| $150,000 or more | 30 | >9 | 0.00 | [-0.20, 0.20] | 0.975 |
| $150,000 or more | 7 | >1 | -0.11 | [-0.32, 0.10] | 0.315 |
| Income Unknown | 30 | >2 | 0.10 | [-0.04, 0.23] | 0.162 |
| Income Unknown | 30 | >3 | 0.09 | [-0.05, 0.23] | 0.187 |
| Income Unknown | 30 | >9 | 0.10 | [-0.05, 0.25] | 0.174 |
| Income Unknown | 7 | >1 | -0.01 | [-0.16, 0.15] | 0.924 |
| Race Other | 30 | >2 | 0.03 | [-0.10, 0.16] | 0.661 |
| Race Other | 30 | >3 | 0.02 | [-0.11, 0.16] | 0.749 |
| Race Other | 30 | >9 | 0.01 | [-0.13, 0.15] | 0.883 |
| Race Other | 7 | >1 | -0.03 | [-0.18, 0.12] | 0.698 |
| Race Unknown | 30 | >2 | 0.17 | [0.04, 0.29] | 0.012 |
| Race Unknown | 30 | >3 | 0.16 | [0.03, 0.29] | 0.015 |
| Race Unknown | 30 | >9 | 0.16 | [0.02, 0.30] | 0.023 |
| Race Unknown | 7 | >1 | 0.11 | [-0.04, 0.25] | 0.139 |
| T1 Distress | 30 | >2 | -0.08 | [-0.22, 0.05] | 0.236 |
| T1 Distress | 30 | >3 | -0.08 | [-0.22, 0.05] | 0.228 |
| T1 Distress | 30 | >9 | -0.14 | [-0.28, 0.00] | 0.054 |
| T1 Distress | 7 | >1 | -0.05 | [-0.20, 0.10] | 0.518 |

*Note.* Active Proportion = the proportion of active meditation practice divided by the overall number of practices. *β* = standardized coefficient. For 30 days of engagement, the sample size when examining >2 practices *n* = 240, >3 practices *n* = 231, >9 practices *n* = 207. When examining just the first seven days of engagement for individuals who engaged in >1 practice, *n* = 203.

**Supplemental Table 8**

Predicting Active Proportion by Demographics for Sample 2 Covarying Total Days of Engagement

| **Demographic Variable** | **Number of Days of App Engagement** | **Number of Practice Sessions** | ***β*** | **95% CI** | ***p*** |
| --- | --- | --- | --- | --- | --- |
| Woman | 30 | >1 | 0.03 | [-0.10, 0.16] | 0.672 |
| Gender Unknown | 30 | >1 | -0.06 | [-0.19, 0.07] | 0.399 |
| Age | 30 | >1 | -0.06 | [-0.19, 0.08] | 0.411 |
| College Grad or Higher | 30 | >1 | -0.03 | [-0.16, 0.11] | 0.709 |
| Education Unknown | 30 | >1 | -0.07 | [-0.20, 0.05] | 0.252 |
| Married or Domestic Partnership | 30 | >1 | 0.04 | [-0.12, 0.20] | 0.610 |
| Marital Status Unknown | 30 | >1 | -0.02 | [-0.15, 0.11] | 0.712 |
| Race Other | 30 | >1 | 0.03 | [-0.10, 0.16] | 0.622 |
| Race Unknown | 30 | >1 | 0.16 | [0.04, 0.29] | 0.011 |
| $50,000-$100,000 | 30 | >1 | 0.19 | [-0.02, 0.40] | 0.080 |
| $100,000-$150,000 | 30 | >1 | 0.18 | [-0.05, 0.42] | 0.120 |
| $150,000 or more | 30 | >1 | 0.03 | [-0.15, 0.22] | 0.719 |
| Income Unknown | 30 | >1 | 0.08 | [-0.05, 0.22] | 0.227 |
| T1 Distress | 30 | >1 | -0.07 | [-0.20, 0.06] | 0.307 |
| Sum Days | 30 | >1 | 0.14 | [0.01, 0.26] | 0.038 |

*Note.* Active Proportion = the proportion of active meditation practice divided by the overall number of practices. *β* = standardized coefficient. For 30 days of engagement, the sample size when examining >1 practices was 248.

**Supplemental Table 9**

Sensitivity Analyses Predicting Utilization and Clinical Outcomes from Active Proportion in Sample 2

| **Outcome Variable** | **Active Proportion Variable** | **Number of Days of App Engagement** | **Number of Practices** | ***β*** | **95% CI** | ***p*** |
| --- | --- | --- | --- | --- | --- | --- |
| Sum Practice | Active Proportion | 30 | >2 | 0.68 | [0.26, 1.10] | 0.001 |
| Sum Practice | Active Proportion^2^ | 30 | >2 | -0.53 | [-0.94, -0.11] | 0.013 |
| Sum Practice | Active Proportion | 30 | >3 | 0.61 | [0.18, 1.05] | 0.006 |
| Sum Practice | Active Proportion^2^ | 30 | >3 | -0.50 | [-0.93, -0.07] | 0.024 |
| Sum Practice | Active Proportion | 30 | >9 | 0.10 | [-0.39, 0.59] | 0.681 |
| Sum Practice | Active Proportion^2^ | 30 | >9 | -0.01 | [-0.49, 0.48] | 0.980 |
| Sum Practice | Active Proportion | 7 | >1 | 0.30 | [-0.11, 0.71] | 0.150 |
| Sum Practice | Active Proportion^2^ | 7 | >1 | -0.30 | [-0.70, 0.11] | 0.154 |
| Sum Days | Active Proportion | 30 | >2 | 0.64 | [0.23, 1.05] | 0.002 |
| Sum Days | Active Proportion^2^ | 30 | >2 | -0.51 | [-0.92, -0.10] | 0.014 |
| Sum Days | Active Proportion | 30 | >3 | 0.58 | [0.15, 1.01] | 0.008 |
| Sum Days | Active Proportion^2^ | 30 | >3 | -0.49 | [-0.92, -0.07] | 0.023 |
| Sum Days | Active Proportion | 30 | >9 | 0.23 | [-0.24, 0.70] | 0.339 |
| Sum Days | Active Proportion^2^ | 30 | >9 | -0.16 | [-0.64, 0.31] | 0.493 |
| Sum Days | Active Proportion | 7 | >1 | -0.10 | [-0.49, 0.30] | 0.630 |
| Sum Days | Active Proportion^2^ | 7 | >1 | 0.02 | [-0.37, 0.42] | 0.903 |
| T2 Psychological Distress | Active Proportion | 30 | >2 | 0.38 | [0.03, 0.73] | 0.035 |
| T2 Psychological Distress | Active Proportion^2^ | 30 | >2 | -0.33 | [-0.68, 0.02] | 0.068 |
| T2 Psychological Distress | Active Proportion | 30 | >3 | 0.36 | [0.01, 0.72] | 0.047 |
| T2 Psychological Distress | Active Proportion^2^ | 30 | >3 | -0.31 | [-0.67, 0.04] | 0.086 |
| T2 Psychological Distress | Active Proportion | 30 | >9 | 0.48 | [0.08, 0.87] | 0.017 |
| T2 Psychological Distress | Active Proportion^2^ | 30 | >9 | -0.43 | [-0.82, -0.04] | 0.033 |
| T2 Psychological Distress | Active Proportion | 7 | >1 | 0.01 | [-0.33, 0.35] | 0.953 |
| T2 Psychological Distress | Active Proportion^2^ | 7 | >1 | 0.01 | [-0.32, 0.35] | 0.941 |
| T3 Psychological Distress | Active Proportion | 30 | >2 | 0.38 | [0.05, 0.71] | 0.026 |
| T3 Psychological Distress | Active Proportion^2^ | 30 | >2 | -0.42 | [-0.75, -0.09] | 0.013 |
| T3 Psychological Distress | Active Proportion | 30 | >3 | 0.45 | [0.11, 0.79] | 0.009 |
| T3 Psychological Distress | Active Proportion^2^ | 30 | >3 | -0.48 | [-0.82, -0.15] | 0.005 |
| T3 Psychological Distress | Active Proportion | 30 | >9 | 0.58 | [0.23, 0.94] | 0.002 |
| T3 Psychological Distress | Active Proportion^2^ | 30 | >9 | -0.58 | [-0.93, -0.22] | 0.002 |
| T3 Psychological Distress | Active Proportion | 7 | >1 | 0.15 | [-0.17, 0.47] | 0.357 |
| T3 Psychological Distress | Active Proportion^2^ | 7 | >1 | -0.26 | [-0.59, 0.06] | 0.105 |

*Note.* Active Proportion = the proportion of active meditation practice divided by the overall number of practices. *β* = standardized coefficient. For 30 days of engagement, the sample size when examining >2 practices was *n* = 240 For >3 practices *n* = 231, >9 practices *n* = 207. When examining just the first seven days of engagement for individuals who engaged in >1 practice, *n* = 203. Demographics and T1 Psychological Distress were included as covariates in all models.

**Supplemental Table 10**

Sensitivity Analyses Predicting Disaggregated Clinical Outcomes from Active Proportion in Sample 2

| **Outcome Variable** | **Active Proportion Variable** | **Number of Days of App Engagement** | **Number of Practices** | ***β*** | **95% CI** | ***p*** |
| --- | --- | --- | --- | --- | --- | --- |
| T2 Anxiety | Active Proportion | 30 | >1 | 0.48 | [0.13, 0.83] | 0.008 |
| T2 Anxiety | Active Proportion^2^ | 30 | >1 | -0.44 | [-0.80, -0.09] | 0.014 |
| T2 Depression | Active Proportion | 30 | >1 | 0.37 | [0.01, 0.73] | 0.042 |
| T2 Depression | Active Proportion^2^ | 30 | >1 | -0.35 | [-0.70, 0.01] | 0.059 |
| T2 Stress | Active Proportion | 30 | >1 | 0.07 | [-0.27, 0.42] | 0.669 |
| T2 Stress | Active Proportion^2^ | 30 | >1 | -0.02 | [-0.36, 0.33] | 0.928 |
| T3 Anxiety | Active Proportion | 30 | >1 | 0.46 | [0.12, 0.80] | 0.009 |
| T3 Anxiety | Active Proportion^2^ | 30 | >1 | -0.51 | [-0.85, -0.17] | 0.004 |
| T3 Depression | Active Proportion | 30 | >1 | 0.37 | [0.01, 0.73] | 0.041 |
| T3 Depression | Active Proportion^2^ | 30 | >1 | -0.38 | [-0.74, -0.02] | 0.038 |
| T3 Stress | Active Proportion | 30 | >1 | 0.11 | [-0.24, 0.45] | 0.537 |
| T3 Stress | Active Proportion^2^ | 30 | >1 | -0.19 | [-0.54, 0.15] | 0.270 |

*Note.* Active Proportion = the proportion of active meditation practice divided by the overall number of practices. *β* = standardized coefficient. Demographics and the respective T1 variable (e.g., T1 Anxiety for T2 and T3 Anxiety models) were included as covariates in all models. For 30 days of engagement, the sample size when examining >1 practices was 248.

**Supplemental Figure 1**

*Association between Sum Practice and Active Proportion in Sample 1*

*Note.* Figures display linear regression line (green) and local regression (i.e., loess) curve (orange). The sample size when examining >1 practice was 26,532. For >2 practices *n* = 21,675, >3 practices *n* = 18,014, >9 practices *n* = 8,345.

**Supplemental Figure 2**

*Association between Sum Days and Active Proportion in Sample 1*

*Note.* Figures display linear regression line (green) and local regression (i.e., loess) curve (orange). The sample size when examining >1 practice was 26,532. For >2 practices *n* = 21,675, >3 practices *n* = 18,014, >9 practices *n* = 8,345.

**Supplemental Figure 3**

*Association between Sum Practice and Active Proportion in Sample 2*

*Note.* Figures display linear regression line (green) and local regression (i.e., loess) curve (orange). The sample size when examining >1 practice was 248. For >2 practices *n* = 240, >3 practices *n* = 231, >9 practices *n* = 207.

**Supplemental Figure 4**

*Association between Sum Days and Active Proportion in Sample 2*

*Note.* Figures display linear regression line (green) and local regression (i.e., loess) curve (orange). The sample size when examining >1 practice was 248. For >2 practices *n* = 240, >3 practices *n* = 231, >9 practices *n* = 207.

**Supplemental Figure 5**

*Association between T2 Psychological Distress and Active Proportion in Sample 2*

*Note.* Figures display linear regression line (green) and local regression (i.e., loess) curve (orange). The sample size when examining >1 practice was 248. For >2 practices *n* = 240, >3 practices *n* = 231, >9 practices *n* = 207. T2 Psychological Distress reflects residualized change scores which were calculated by taking the difference between an individual's observed post-test score and their predicted post-test score based on their pre-test score (i.e., lower residualized change scores indicate greater reductions in distress).

**Supplemental Figure 6**

*Association between T3 Psychological Distress and Active Proportion in Sample 2*

*Note.* Figures display linear regression line (green) and local regression (i.e., loess) curve (orange). The sample size when examining >1 practice was 248. For >2 practices *n* = 240, >3 practices *n* = 231, >9 practices *n* = 207. T3 Psychological Distress reflects residualized change scores which were calculated by taking the difference between an individual's observed follow-up score and their predicted follow-up score based on their pre-test score (i.e., lower residualized change scores indicate greater reductions in distress).
